# Metabolically-targeted dCas9 expression in bacteria

**DOI:** 10.1093/nar/gkac1248

**Published:** 2023-01-11

**Authors:** Gregory M Pellegrino, Tyler S Browne, Keerthana Sharath, Khaleda A Bari, Sarah J Vancuren, Emma Allen-Vercoe, Gregory B Gloor, David R Edgell

**Affiliations:** Schulich School of Medicine and Dentistry, Department of Biochemistry, London, Ontario N6A 5C1, Canada; Schulich School of Medicine and Dentistry, Department of Biochemistry, London, Ontario N6A 5C1, Canada; Schulich School of Medicine and Dentistry, Department of Biochemistry, London, Ontario N6A 5C1, Canada; Schulich School of Medicine and Dentistry, Department of Biochemistry, London, Ontario N6A 5C1, Canada; Department of Molecular and Cellular Biology, University of Guelph, Guelph, Ontario N1G 2W1, Canada; Department of Molecular and Cellular Biology, University of Guelph, Guelph, Ontario N1G 2W1, Canada; Schulich School of Medicine and Dentistry, Department of Biochemistry, London, Ontario N6A 5C1, Canada; Schulich School of Medicine and Dentistry, Department of Biochemistry, London, Ontario N6A 5C1, Canada

## Abstract

The ability to restrict gene expression to a relevant bacterial species in a complex microbiome is an unsolved problem. In the context of the human microbiome, one desirable target metabolic activity are glucuronide-utilization enzymes (GUS) that are implicated in the toxic re-activation of glucuronidated compounds in the human gastrointestinal (GI) tract, including the chemotherapeutic drug irinotecan. Here, we take advantage of the variable distribution of GUS enzymes in bacteria as a means to distinguish between bacteria with GUS activity, and re-purpose the glucuronide-responsive GusR transcription factor as a biosensor to regulate dCas9 expression in response to glucuronide inducers. We fused the *Escherichia coli gusA* regulatory region to the dCas9 gene to create pGreg-dCas9, and showed that dCas9 expression is induced by glucuronides, but not other carbon sources. When conjugated from *E. coli* to Gammaproteobacteria derived from human stool, dCas9 expression from pGreg-dCas9 was restricted to GUS-positive bacteria. dCas9-sgRNAs targeted to *gusA* specifically down-regulated *gus* operon transcription in Gammaproteobacteria, with a resulting ∼100-fold decrease in GusA activity. Our data outline a general strategy to re-purpose bacterial transcription factors responsive to exogenous metabolites for precise ligand-dependent expression of genetic tools such as dCas9 in diverse bacterial species.

## INTRODUCTION

The bacterial Clustered Regularly Interspaced Palindromic Repeat (CRISPR) system and its associated proteins (Cas) are widely used for gene-editing applications (1–3). In bacteria, the Cas9 proteins from *Streptococcus pyogenes* (SpCas9) and *Staphylococcus aureus* (SaCas9) can be employed as novel antimicrobial agents to modulate the activity and composition of microbial communities by eliminating specific bacteria through Cas-mediated cleavage of the bacterial chromosome (4–9). Alternatively, catalytically inactive Cas9 variants (‘dead’ Cas9 or dCas9) can down-regulate the expression of targeted genes by interfering with transcription in a strategy referred to as CRISPR interference (CRISPRi) (1,10). This strategy is useful when the goal is to modulate bacterial gene expression and metabolic activity rather than to eliminate specific bacteria, as would be the case with active Cas9.

The microbiome of the human gastrointestinal (GI) tract can exert profound influences on health and disease through changes in microbial composition or metabolic activity (11–18). A key concept regarding metabolism in microbiomes is that the relevant metabolic pathways are variably distributed and only active in a subset of bacteria (19,20). Moreover, key metabolically-relevant bacteria can be members of a consortia of bacteria that exchange metabolites, but also have differential abundance and expression of specific genes (21–23). Because expression of dCas9 in bacteria can be toxic (24–26), it would be advantageous to limit dCas9 expression to metabolically-relevant bacteria so that off-target effects and fitness costs are eliminated in non-relevant bacteria. This approach would also minimize the occurrence of counter selection and mutational inactivation of dCas9. While a number of studies have shown that dCas9 can modulate gene expression in microbiomes, either by genome engineering of key species (27–29) or by introduction of episomal- or phage-based systems (30), current strategies cannot link dCas9 activity to metabolic pathways that are the intended target of down-regulation. Here, we propose that the variable occurrence of metabolic pathways in bacteria can be used as a means to restrict dCas9 activity to metabolically-relevant species.

**Figure 1.**
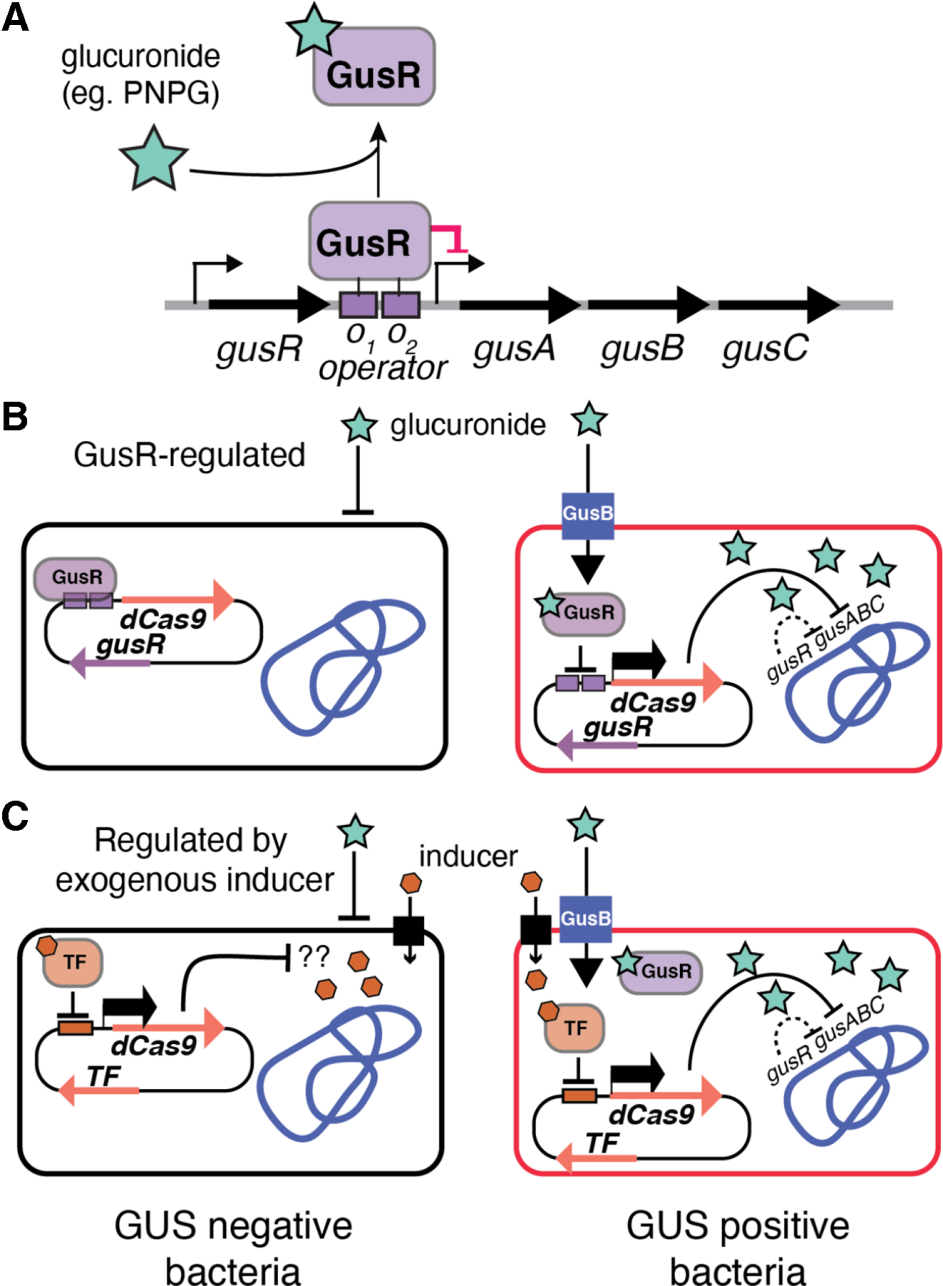
Strategy to regulate dCas9 with ligand-responsive transcription factors. (**A**) Schematic of the *E. coli gusA* operon (not to scale) and impact of glucuronide on GusR binding and *gus* expression. Small right-facing arrows indicate promoters and large right-facing arrows indicate individual genes. The GusR operator binding sites (*O_1_* and *O_2_*) upstream of GusA are indicated by filled rectangles. (**B**) Strategy to regulate dCas9 expression using GusR regulation and glucuronide or (**C**) an exogenous inducer (polygon) and associated transcription factor (TF). Glucuronide (green star) can be imported to induce GusR-regulated dCas9 expression only in GUS+ bacteria (top right, red outlined bacteria), but not in GUS− negative bacteria (top left, black outlined bacteria). In contrast, dCas9 expression will be induced by an exogenous compound (such as l-arabinose) in both GUS+ or GUS− negative bacteria.

In the context of the human microbiome, one relevant metabolic activity that has a variable bacterial distribution is β-glucuronidase (GUS) enzymes in Psuedomonadota, Bacillota and Bacteroidota that cleave glucuronic acid from a variety of compounds for use as a carbon source (31–34). In *Escherichia coli* and other Psuedomonadota, three genes are found in the *gus* operon (also called *uid*); *gusA* encoding the β-glucuronidase, *gusB* encoding the glucuronide transporter, and *gusC* encoding a membrane-associated protein that enhances the transport activity of GusB (35,36,36–39) (Figure 1A). Gus operon expression is negatively regulated by binding of the TetR/lysR family GusR transcriptional regulator to operator sequences upstream of *gusA*, and induced by the presence of glucuronides that bind GusR to prevent DNA binding and relieve repression (38,39,39–45). Compounds that are substrates for GUS enzymes include xenobiotic medications that have been glucuronidated by mammalian UDP-glucuronosyltransferases (46). In particular, the anti-cancer drug irinotecan (CPT-11) that is activated by carboxylesterases to SN-38 and then glucuronidated to SN-38G is a substrate for GUS enzymes (47). Microbial GUS cleavage of SN-38G results in localized reactivation of the active form (SN-38), causing dose-limiting toxicity and intestinal wall shedding, which is a particularly serious side-effect of this class of anti-neoplastic drugs (48). Small molecule inhibitors designed to inactivate a subset of GUS enzymes have shown promise in knocking down GUS activity in mouse models (49,50).

Here, we develop a strategy to restrict dCas9 activity to relevant bacteria by genetically linking its expression to the metabolic pathway that is the intended target of down regulation, and use the GUS system as proof-of-principle. We genetically coupled dCas9 to the *gusA* promoter region that is negatively regulated by GusR (Figure 1B) and show that dCas9 is expressed only in the presence of exogenous glucuronides and not other carbon sources. Furthermore, *gus*-regulated dCas9 is expressed only in GUS-positive bacteria and specifically down-regulates both *gus* operon transcription and GusA activity. Our study highlights the utility of dCas9 as a means to repress potentially toxic GUS activity in the human microbiome. Our data suggest a general strategy to re-purpose metabolite-regulated bacterial transcription factors to control dCas9 expression for synthetic biology applications in targeted bacterial strains within microbial communities.

## MATERIALS AND METHODS

### Bacterial and yeast strains

*E. coli* EPI300 (F′ λ^−^*mcrA* Δ(*mrr-hsdRMS-mcrBC*) ϕ80d*lacZ*Δ*M15* Δ*(lac)X74 recA1 endA1 araD139* Δ*(ara, leu)7697 galU galK rpsL* (Str^*R*^) *nupG trfA dhfr*) (Epicenter) was used for cloning and maintenance of plasmids. *E. coli* ECGE101 Δ*dapA* is an auxotrophic strain of EPI300 requiring diaminopimelic acid (DAP) supplementation (0.3 mM) and was used as a conjugative donor (51). *E. coli* BL21(DE3) (str. B F′ *ompT* *gal* *dcm* *lon* *hsdS*_*B*_(*r*_*B*_^−^*m*_*B*_^−^) λ(DE3 [*lacI lacUV5-T7p07 ind1 sam7 nin5*]) was used for β-glucuronidase assays. *E. coli* (F′ λ^−^*mcrA* Δ(*mrr-hsdRMS-mcrBC*) ϕ80d*lacZ*Δ*M15* Δ*(lac)X74 recA1 endA1 araD139* Δ*(ara, leu)7697 galU galK rpsL* (Str^*R*^) *nupG trfA dhfr*) Δ(*gusA*) (acquired from Dr. Bogumil Karas at Western University) was used as a negative control for chromogenic and fluorescent assays. All further bacterial strains were tested for natural antibiotic resistances on LB agar plates containing one of the following: 25 µg/ml chloramphenicol, 40 µg/ml gentamycin, 100 µg/ml ampicillin, 50 µg/ml kanamycin, or 20 µg/ml tetracycline. *Salmonella enterica* subsp. *enterica* serovar Typhimurium strain LT2 (Δ*hilA*::Kan^R^) (acquired from Dr. David Haniford at Western University) was used as a *gusA*-negative recipient strain. *E. coli* strain AC2811 (Amp^R^) and *E. coli* strain OBEAV1 (Amp^R^ and Tet^R^) were isolated from patient-derived samples by the Allen-Vercoe lab. *Shigella sonnei* (NCDC 1120-66 [CIP 104223], derived from ATCC strain 25931) and *S. sonnei* (WRAIR I virulent, derived from ATCC strain 29930, Tet^R^) were purchased in KWIK-STIK^TM^ format from Micronostyx Inc. and cultured per manufacturers instructions. *Saccharomyces cerevisiae* VL6-48 (*MAT***a**, *his3Δ200, trpΔ1, ura3-52, ade2-101, lys2, psi + cir*°) was used for yeast assembly of plasmids.

### Plasmid construction

A list of primers is provided in Supplementary Table S3. The pBAD-dCas9 plasmid was constructed by polymerase chain reaction (PCR) amplification of DNA fragments with 60-120 bp homology overlaps from pre-existing plasmids. The *oriT* fragment was amplified from pPtGE30 (52) using primers DE3302 and DE3303. The *araC* gene and pBAD promoter were amplified from pBAD-24 (53) using primers DE3304 and DE3305. The CEN6-ARSH4-HIS3 yeast element was amplified from pPtGE30 (52) using primers DE3316 and DE3351. The p15A origin and chloramphenicol acetyl-transferase gene were amplified using primers DE3309 and DE3352 from a modified pX458 plasmid (54).The dCas9 gene was amplified from a modified pX458 plasmid using primers DE3306 and DE3307. The sgRNA DNA fragment was amplified from a modified pX458 plasmid using primers 3308 and 3315. A modified yeast assembly protocol (4) was used to assemble the above PCR fragments to create the pBAD-dCas9 plasmid. The pGreg-GusR-dCas9 plasmid was constructed by PCR amplification of the pBAD-dCas9 plasmid using primers DE5317 and DE5339, which removed the *araC* gene and the pBAD promoter. Genomic DNA was isolated from *E. coli* BL21(DE3) using the Monarch Genomic DNA Purification Kit, and the *gusR* gene and GUS operon promoter were amplified from the gDNA using primers DE5275 and DE5338. The two fragments were assembled together with the modified yeast assembly protocol. A HA-tag was added to the C-terminal end of the *gusR* gene with primers DE5753 and DE5754. The pGreg-dCas9 plasmid was constructed by PCR amplification of the pGreg-GusR-dCas9 plasmid with primers DE5489 and DE5490 to remove the *gusR* gene and its promoter while leaving the GUS operon promoter driving dCas9 expression intact. A blunt-end ligation with T4 DNA ligase was done to recircularize the linear PCR product. The pGreg-GusR-dCas9 plasmid was PCR amplified to remove the *gusR* promoter, ribosome binding site, and start codon with primers DE6215 and DE6216. AP1-AP11 were ordered as gBlocks from Integrated DNA Technologies (IDT) containing alternate promoters, start codons, and/or ribosome binding sites with 20-30 bp homology to the PCR product, which were then inserted into the plasmid with the NEBuilder HiFi DNA assembly kit following the manufacturers instructions to create plasmids pGreg-GusR-dCas9.1-pGreg-GusR-dCas9.11, respectively. The original pGreg-GusR-dCas9 plasmid was then referred to as pGreg-GusR-dCas9.0 to distinguish it from the alternate *gusR* promoters. Transformants were screened by outgrowth in LB supplemented with 1 mM pNPG (4-nitrophenyl-β-D glucuronide, Sigma-Aldrich) at 37°C with 548 cpm continous double orbital shaking while measuring *A*_410_ in the BioTek Epoch 2 microplate spectrophotometer to determine which clones had recovered GusA activity (Supplementary Figure S8B). Alternate promoter clones with higher *A*_410_ relative to pGreg-GusR-dCas9.0 were verified through Sanger sequencing with primers DE6115 and DE6116 (London Regional Genomics Centre). Primer DE5992 paired with DE6279 and primer DE5993 paired with DE6280 were used to PCR amplify the pGreg-GusR-dCas9.7 plasmid in two fragments, removing the GusR O_1_ site. The two fragments were assembled using the NEBuilder HiFi DNA assembly kit to create the OpΔ plasmid. Deletions were screened by PCR with primers DE3537 and DE5489, then a correct clone was further verified through Sanger sequencing (London Regional Genomics Centre).

### sgRNA cloning

Single strand DNA oligonucleotides were ordered from IDT: top strands with sequences 5’-CACGN_20_G-3’ and bottom strands with sequences 5’-AAAACN_20_-3’ (N_20_ refers to the crRNA sequence). Complementary top and bottom strands were annealed and phosphorylated. The duplexed strands were then cloned into dCas9 plasmids via Golden Gate assembly (55) with BsaI-HF v2 (NEB). Correct sgRNA insertion was verified through PCR screens with primers DuetUp2 and T7 Rev, then correct clones were further verified by Sanger sequencing (London Regional Genomics Centre). The sgRNA array was designed with an extra DNA spacer containing primer sites and BsaI cut sites on both ends so it could be cloned into the dCas9 plasmids with Golden Gate assembly. The sgRNA array (746 bps) was ordered from Codex DNA, Inc. and printed on a BioXP™ 3200 DNA printer. The fragment was PCR amplified with primers DuetUp2 and T7 Rev, then cloned and verified as described for the sgRNAs.

### Bacterial conjugation

pBAD-dCas9 plasmids and pGreg-GusR-dCas9.7 plasmids with sgRNA_349_ or no guide were transformed into *E. coli* ECGE101 Δ*dapA* cells harbouring the conjugative pTA-Mob helper plasmid (56). Saturated cultures of donor *E. coli* ECGE101 Δ*dapA* with dCas9 plasmids and saturated cultures of the recipient strains were diluted 1:25 and 1:100 respectively into non-selective LB (10 g/l tryptone, 5 g/l yeast extract, 10 g/l NaCl) supplemented with 0.2% d-glucose and 0.3 mM DAP and grown to an OD_600_ of 0.5. Donor cultures (200 µl each) were mixed with 200 µl of each recipient strain on a non-selective LB plate supplemented with 0.2% d-glucose and 0.3 mM DAP. Plates were incubated 1 h at 37°C for conjugation. Agar plates were then scraped with 500 µl of SOC media (20 g/l tryptone, 5 g/l yeast extract, 0.5 g/l NaCl, 2.5 mM KCl, 10 mM MgCl_2_, and 0.2% d-glucose) supplemented with DAP. Resulting cell suspensions were serially diluted in SOC media and plated on LB agar plates supplemented with 25 µg/ml chloramphenicol and 0.2% d-glucose to select for exconjugants. Three exconjugant colonies for each strain with each plasmid were selected for chromogenic assays; a single exconjugant colony from each conjugation was used for western blots and fluorescent assays.

### β-Glucuronidase activity assays

For determination of GUS-status on solid media, cultures of each strain were spot-plated onto LB agar plates supplemented with 40 µg/ml X-Gluc (5-bromo-4-chloro-3-indolyl-β-d-glucuronic acid, BioBasic Canada) and incubated at 37°C overnight. For assays using liquid cultures, overnight cultures were diluted 1:50 into selective LB media supplemented with 1 mM pNPG or methyl-β-d-glucuronide sodium salt (MetGluc, Sigma-Aldrich). Cultures were grown at 37°C, 225 rpm shaking for 3 h. For pNPG induced cultures, 1 mL of each culture was transferred to a microcentrifuge tube and centrifuged for 10 min at 13 000 rpm. The supernatant was decanted and the cell pellet resuspended in 1 ml of LB media supplemented with 25 µg/ml chloramphenicol. The resuspended cells were then centrifuged, decanted, and resuspended again. For MetGluc induced cultures, cells were not centrifuged and resuspended. Instead, 1 ml was aliquoted into an Eppendorf tube for further steps. 100 µl of each resuspension was pipetted into a clear-bottom 96-well plate and OD_600_ was measured in a BioTek Epoch 2 Microplate Spectrophotometer. To permeabilize cells, 30 µl of 0.1% SDS and 60 µl of chloroform were added to each resuspension and vortexed for 30 s. For chromogenic assays, 20 µl of permeabilized cells were mixed with 80 µl of reaction buffer (150 mM NaCl, 20 mM HEPES, pH 7.5, pNPG added to 1.25 mM immediately prior to use) pre-warmed to 37°C in a clear-bottom 96-well plate. Absorbance at 410 nm was measured every minute for 1 h at 37°C with 548 cpm continuous double orbital shaking in the BioTek Epoch 2 microplate spectrophotometer. GusA units were calculated by multiplying the slope of the absorbance at 410 nm/min by 1000 and then dividing that value by the volume of cells (0.02 ml) multiplied by the OD_600_ of the culture. For fluorometric assays, 10 µl of permeabilized cells were added to 90 µl of reaction buffer (50 mM NaCl, 20 mM HEPES, pH 7.4, SN38-G added to 0.15 mM immediately prior to measurement) in a black clear-bottom 96-well plate. Using an excitation of 250 nm, emission at 420 nm was measured every minute for 2 h at 37°C with 548 cpm continuous double orbital shaking in a BioTek Synergy H1 microplate reader using the bottom optics position with auto gain. The rate of SN-38G hydrolysis was calculated by multiplying the slope of the fluorescence at 420 nm/min by -1 and then dividing that value by the volume of cells (0.01 ml) multiplied by the OD_600_ of the culture.

### Total RNA Preparation

Three biological replicates were performed from three separate transformations of pBAD-dCas9 plasmids into *E. coli* BL21(DE3). A single colony was picked from a streak plate and grown to saturation overnight in LB media supplemented with 25 µg/ml chloramphenicol and 0.2% D-glucose, and diluted 1:50 into LB media supplemented with 1 mM pNPG and 25 µg/ml chloramphenicol. Cultures were grown at 37°C, 225 rpm shaking for 2.5 h, at which point 1 ml of culture was centrifuged at 16 000 g for 5 minutes at 4°C. The cell pellet was resuspended in 1 ml of 4°C Trizol reagent (Invitrogen). Samples were incubated at 65°C for 10 min, 0.2 ml of chloroform added and mixed by inverting for 15 s, then incubated at room temperature for 3 min. Samples were centrifuged at 16 000 g for 5 min at 4°C and the upper aqueous phase was transferred to a new tube with ∼1 volume of ethanol. Samples were loaded into a RNA cleanup column (Monarch RNA Cleanup Kit) and centrifuged at 16 000 g for 1 minute at room temperature. Columns were washed twice with 0.5 ml of RNA cleanup wash buffer at 16 000 g for 1 min at room temperature. Columns were spun at 16 000 g for 1 min to ensure the column was dry, and the RNA was eluted with 50 µl nuclease-free water at 16 000 g for 1 min.

### RNA-Seq

Total RNA samples were sent to the London Regional Genomics Centre for RNA-sequencing. The RNA quality was assessed using the Agilent 2100 Bioanalyzer, and then rRNA reduction was performed and indexed libraries were created. The libraries were sequenced using the Illumina NextSeq High Output 75 cycle sequencing kit with single end reads. Reads were trimmed with Trimmomatic version 0.36 with options LEADING:10 TRAILING:10 (57). Processed reads were mapped to genome and plasmid reference sequences using Hisat2 version 2.2.0 (58). Htseq-count version 0.13.5 was used to count the number of reads mapping to each annotated feature within the genome and plasmid (59). DESeq2 version 1.32.0 was used to detect differentially expressed genes between the sgRNA-containing strains and the no guide strain using a Wald test with the default false discovery rate of 0.1 (60). Scripts for processing RNAseq data are available at https://github.com/tbrowne5/Metabolically-targeted-dCas9-expression-in-bacteria-.git.

### Western blotting

Samples were resolved on SDS-polyacrylamide gels (8% for dCas9/GusA blots or 15% for GusR blots) and electroblotted to a polyvinylidene difluoride (PVDF) membrane using a Trans-Blot Turbo Transfer System (Bio-Rad). All incubations and washes were performed with gentle agitation on a rocking platform. Membranes were incubated for 1 h in blocking solution (3% bovine serum albumin (BSA), 0.1% Tween-20, Tris-buffered saline (TBS: 150 mM NaCl, 50 mM Tris–HCl, pH 7.5)) before adding primary antibody at a 1:1000 final dilution. Anti-Cas9 rabbit polyclonal primary antibody (Rockland, 600-401-GK0S) was used for dCas9 blots, anti-β-glucuronidase N-terminal rabbit polyclonal primary antibody (Sigma-Aldrich, G5420) was used for GusA blots, and anti-HA-tag rabbit monoclonal primary antibody (Invitrogen, MA5-27915) was used for GusR blots. Membranes were incubated overnight at 4°C, washed 3 times for 10 min each (1% BSA, 0.1% Tween-20, TBS), and then incubated with anti-rabbit (Sigma-Aldrich, GENA9340) horseradish peroxidase-linked secondary antibody for 2 h at a 1:5000 final dilution in washing solution. Membranes were then washed in TBS with 0.1% Tween-20 for 10 min three times, followed by one wash for 10 min in TBS. Blots were developed using Clarity ECL western blotting Substrate (Bio-Rad) following the manufacturer’s instructions and imaged with a ChemiDoc XRS+ System (Bio-Rad).

### Mixed culture experiments

Saturated overnight cultures of donor *E. coli* ECGE101 Δ*dapA* with dCas9 plasmids were diluted 1:100 into non-selective LB media supplemented with 0.2% d-glucose and 0.3 mM DAP. Saturated overnight cultures of the recipient strains (*S*. Typhimurium LT2, *E. coli* AC2811, *E. coli* OBEAV1 and *S. sonnei* ATCC 29930) were diluted 1:100, 1:1000, 1:1000 and 1:200, respectively, into non-selective LB media supplemented with 0.2% glucose and 0.3 mM DAP. All cultures were grown at 37°C with 225 rpm shaking for 2.5 hours, until the *A*_600_ was ∼0.5. Each donor culture (200 µl) was mixed with 200 µl of each recipient strain (individually or in a mixed culture) on a non-selective LB plate supplemented with D-glucose and DAP and incubated 1 hour at 37°C for conjugation. Agar plates were then scraped with 500 µl of SOC and 100 µl of the resulting cell suspensions were inoculated into 5 mL of LB supplemented with 25 µg/ml chloramphenicol and 0.2% D-glucose and grown overnight at 37°C with 225 rpm shaking. The remaining cell suspensions were serially diluted in SOC and 10 µl of 10^−2^–10^−^^7^ diluted cells from each conjugation were spotted onto 9 types of LB agar plates to count species in the cultures: chloramphenicol (25 µg/ml), gentamycin (40 µg/ml), and DAP for *E. coli* ECGE101 donors; kanamycin (50 µg/ml) for *S*. Typhimurium LT2 recipients; kanamycin (50 µg/ml) and chloramphenicol (25 µg/ml) for *S*. Typhimurium LT2 exconjugants; ampicillin (100 µg/ml) for *E. coli* AC2811 recipients; ampicillin (100 µg/ml) and chloramphenicol (25 µg/ml) for *E. coli* AC2811 exconjugants; tetracycline (20 µg/ml) for *S. sonnei* ATCC 29930 recipients; tetracycline (20 µg/ml) and chloramphenicol (25 µg/ml) for *S. sonnei* ATCC 29930 exconjugants; ampicillin (100 µg/ml) and tetracycline (20 µg/ml) for *E. coli* OBEAV1 recipients; and ampicillin (100 µg/ml), tetracycline (20 µg/ml) and chloramphenicol (25 µg/ml) for *E. coli* OBEAV1 exconjugants. The plates were incubated overnight at 37°C and colonies were counted the next day to determine colony forming units (CFU)/ml. Overnight cultures were treated as described above for liquid culture chromogenic β-glucuronidase activity assays. At the time of the activity assay, spot-plating was performed again.

## RESULTS

### Glucuronide- and arabinose-regulated dCas9 represses GusA activity

**Figure 2.**
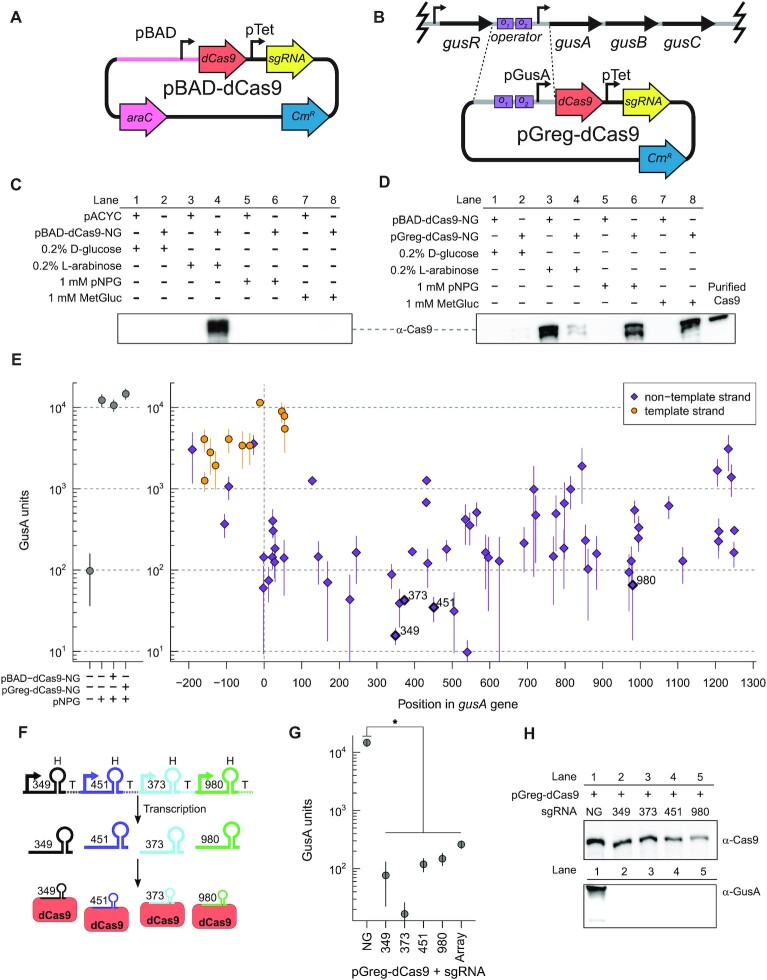
Repression of GusA activity in *E. coli* with dCas9. (**A**) Schematic of the arabinose-regulated pBAD-dCas9. Large right-facing arrows represent genes and small right-facing arrows represent promoters. Cm^R^, chloramphenicol resistance gene; pTet, promoter from the tetracycline resistance gene; pBAD, arabinose-inducible promoter. (**B**) Schematic of pGreg-dCas9 with the *E. coli gus* operon (not to scale) depicted on top with dashed line indicating the *gusA* regulatory region cloned upstream of dCas9. *O_1_* and *O_2_*, GusR operator binding sites; pGusA, promoter from the *gusA* regulatory region. (**C**) Expression of dCas9 from *E. coli* harbouring pBAD-dCas9 under the indicated conditions assessed by western blot with an anti-Cas9 antibody. (**D**) Expression of dCas9 under the indicated conditions with *E. coli* harbouring pBad-dCas9 or pGreg-dCas9 assessed by western blots with an anti-Cas9 antibody. The positive control is 5 ng of purified *Streptococcus pyogenes* Cas9. (**E**) Impact of tiling sgRNAs along the length of the *gusA* gene on GusA activity. Plot of GusA activity (left) in control strains expressing dCas9 without an sgRNA and in the absence or presence of 1 mM pNPG. Plot of GusA activity (right) in dCas9-sgRNA strains for 74 individual sgRNAs targeted to the upstream or coding region of GusA on either the template (orange circles) or non-template (purple diamonds) strand. sgRNAs 349, 373, 451 and 980 used in later experiments are indicated. (**F**) Schematic of sgRNA multi-array to express sgRNA_349_, sgRNA_451_, sgRNA_373_ and sgRNA_980_. Right-facing coloured arrows represent different promoters for each sgRNA. H, sgRNA handle sequence; T, sgRNA terminator sequence. Note that promoter, handle and terminator sequences are different for each sgRNA. (**G**) Knockdown of *E. coli* GusA activity with pGreg-dCas9 and four different sgRNAs, or an array consisting of the same four sgRNAs. (**H**) Western blot of dCas9 and GusA expression in pGreg-dCas9 strains without (NG) or with the indicated sgRNAs. Uncropped images for gel images in panels (C), (D) and (H) are in Supplementary Figures S3, S4 and S5, respectively. For panels (E) and (G) data points are mean values from three biological replicates with whiskers indicating the mean plus or minus the standard deviation. *P < 0.05 calculated by Welch’s *t*-test.

We designed two plasmid-based systems (Figure 2A and B) to examine dCas9 repression of the *gusA* gene based on the contrasting strategies depicted in Figure 1. On the first plasmid, pBAD-dCas9, the pBAD arabinose-regulated promoter was fused to the dCas9 coding region to facilitate dCas9 expression independent of whether bacteria were GUS-positive or -negative (Figure 2A, Supplementary Figure S1A). On the second plasmid, pGreg-dCas9, the *gusA* promoter region that contains the GusR binding sites was fused to the dCas9 coding region (Figure 2B, Supplementary Figure S1B). With pGreg-dCas9, dCas9 should be expressed only in the presence of glucuronides and only in GUS-positive bacteria that contain the appropriate transporter and transcriptional regulator (GusB and GusR, respectively). On both plasmids, a weakly constitutive promoter derived from the tetracycline resistance gene expressed the sgRNA.

We examined dCas9 expression in cell extracts of the GUS-positive *E. coli* BL21(DE3) (Supplementary Figure S2) harbouring pBAD-dCas9 or pGreg-dCas9 by western blots with an anti-Cas9 antibody. With pBAD-dCas9, we observed robust dCas9 expression only in the presence of 0.2% l-arabinose (Figure 2C, lane 4 and Figure 2D, lane 3) and repression in the presence of 0.2% d-glucose (Figure 2C, lane 2 and Figure 2D, lane 1). Addition of either 1 mM pNPG or MetGluc did not induce dCas9 expression from the pBAD promoter (Figure 2D, lanes 5 and 7). With pGreg-dCas9, dCas9 expression was strongly induced by the presence of exogenously added glucuronides; either 1 mM pNPG or 1 mM MetGluc, which are both known to induce GusA expression in *E. coli* (44,61,62) (Figure 2D, lanes 6 and 8). At high expression levels, degradation of dCas9 was observed, consistent with previous reports (25). Addition of higher amounts of pNPG (2-5 mM) to media caused slow growth and were not examined further. Weaker levels of dCas9 expression were observed when 0.2% d-glucose or 0.2% l-arabinose were added to the media (Figure 2D, lanes 2 and 4), consistent with differing effects of the two sugars on catabolite repression of the *gus* operon in *E. coli* (37). Taken together, these results establish plasmid-based systems for regulation of dCas9 expression based on the addition of different exogenous inducers, glucuronide or arabinose.

We next used the plasmid-based systems to examine the impact of 74 different sgRNAs that were tiled along the *gusA* promoter and coding region, targeting the promoter and coding region on both the template and non-template strands based on existing criteria (24,63) (Figure 2E, Supplementary Table S1). sgRNAs are identified by the position relative to the first nucleotide of *gusA*, with negative values indicating binding sites in the upstream promoter region. The impact of sgRNAs on *E. coli* BL21(DE3) GusA activity was measured by chromogenic assays with pNPG (Figure 2E). In the absence of dCas9/sgRNA expression, baseline GusA activity was determined to be ∼100 GusA units under non-inducing conditions (no pNPG addition), whereas addition of 1 mM pNPG induced GusA activity to ∼10000 units with either pGreg-dCas9 or pBAD-dCas9 (Figure 2E, left panel). We consistently observed no detectable pNPG hydrolysis with an *E. coli* Δ*gusA* knockout strain. With pBAD-dCas9, we found that addition of 0.2% L-arabinose or 0.2% D-glucose to media in combination in 1 mM pNPG resulted in low GusA activity (Supplementary Figure S6), likely due to catabolite repression of the *gus* operon by glucose and arabinose. This condition confounded GusA activity measurements with pBAD-dCas9 expressing sgRNAs; all assays were therefore performed under basal conditions (no addition of arabinose). We found that sgRNAs targeted to the non-template strand versus the template strand showed higher levels of GusA repression, with the most active sgRNAs showing ∼100-fold repression (Figure 2E, right panel). sgRNAs targeted within ∼500-bp of the ATG initiation codon had the strongest repressive effect, agreeing with past studies on the optimal positioning of dCas9-sgRNAs for repression of bacterial gene expression (1,24,63).

We also designed a non-repetitive sgRNA array (64) to express the four highly active sgRNAs (349, 373, 451, 980; Figure 2F). The benefit of the sgRNA array would be limiting potential mutational inactivation of any single sgRNA. We performed a paired experiment where we used the same cell extracts of *E. coli* harbouring pGreg-dCas9 expressing the same four single sgRNAs or the array to measure knockdown of GusA enzymatic activity (Figure 2G), and to assess GusA and dCas9 protein levels by western blotting (Figure 2H, Supplementary Figure S5). Notably, we observed robust dCas9 expression and an almost complete knockdown of GusA protein when dCas9 was individually co-expressed from pGreg-dCas9 with four different sgRNAs (349, 373, 451 and 980) (Figure 2H and Supplementary Figure S5), correlating with knockdown of GusA activity (Figure 2G). In contrast, no knockdown of GusA activity or protein levels was observed when dCas9 was expressed without an sgRNA (Figure 2G, NG; Figure 2H, NG; and Supplementary Figure S5). Similar knockdown of GusA protein levels was observed with pBAD-dCas9 expressing the active sgRNA_349_ (Supplementary Figure S6); (Supplementary Figure S7, lanes 7 and 11). In contrast, the poorly active sgRNA_−__11_ was less effective in knocking down GusA protein levels (Supplementary Figure S7, lanes 8 and 12). With the arrayed sgRNAs, we observed ∼56-fold knockdown of GusA activity (Figure 2G), lower than the ∼100-fold knockdown of activity with other single sgRNAs. This data suggests that CRISPRi is an effective strategy to knockdown GusA activity but that knockdown with multiple sgRNAs is not additive.

### RNAseq analyses reveal specific repression of glucuronide utilization genes

**Figure 3.**
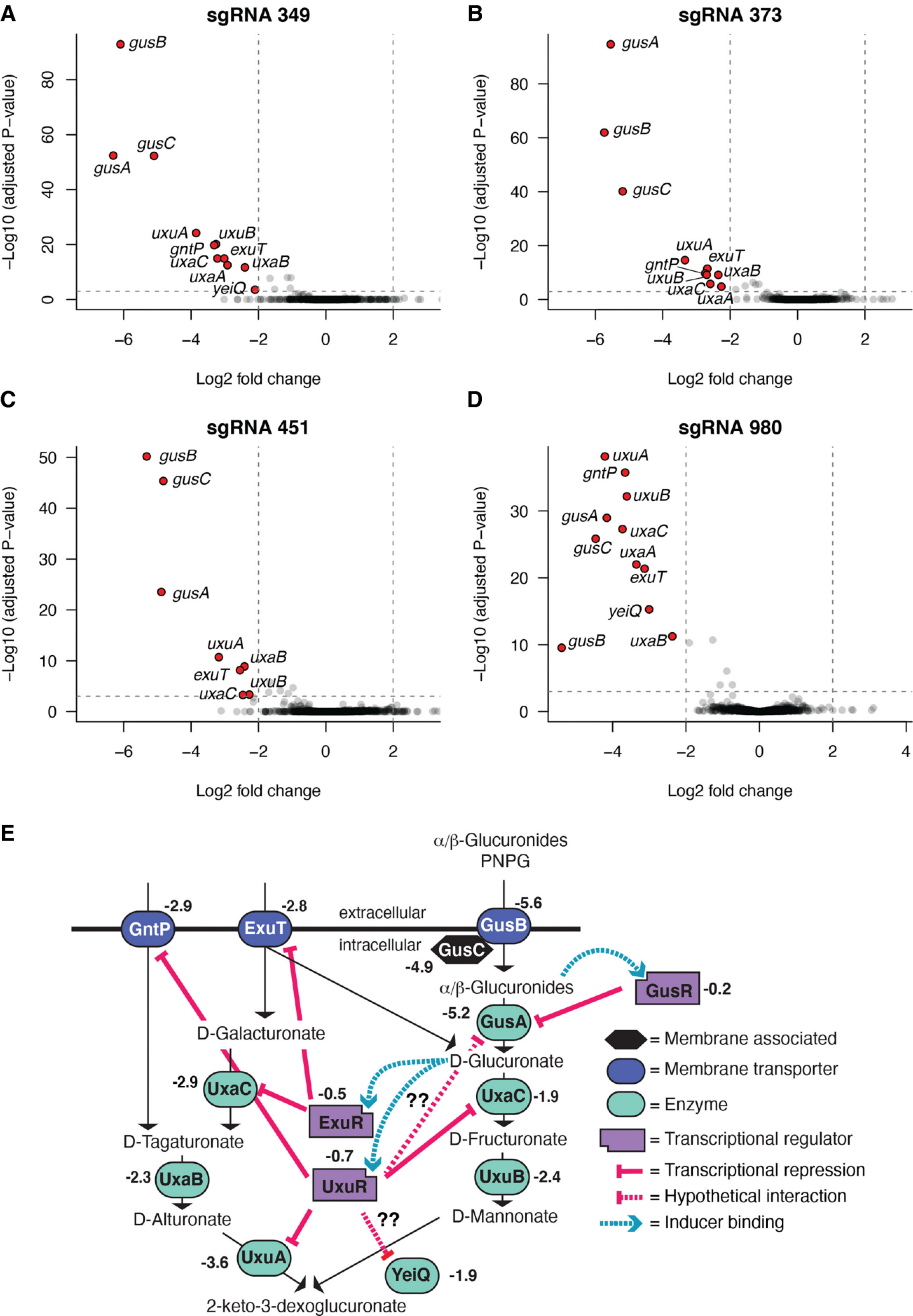
RNAseq analysis of global gene expression changes in *E. coli* strains expressing dCas9 and sgRNAs. (**A**–**D**) Volcano plots of fold changes in gene expression for the indicated sgRNAs. Each point represents a different *E. coli* gene. Grey points have less than 4-fold gene expression change (vertical dashed lines) and are judged not significant (horizontal dashed line, FDR = 1 ×10^-3^). Red points have >4-fold change in expression and are significant. (**E**) Schematic of glucuronide metabolism in *E. coli* (adapted from (65)). The arithmetic mean fold-change in gene expression for all four sgRNAs is indicated beside each protein.

The impact of CRISPRi on *gusA* and global gene expression was examined by RNAseq from strains expressing one of four different sgRNAs (sgRNA_349_, sgRNA_373_, sgRNA_451_, sgRNA_980_) (Supplementary Table S2). With all four individual sgRNAs (Figure 3A–D), we consistently found that transcripts from the *gus* operon (*gusA*, *gusB* and *gusC*) were strongly repressed at least 4-fold relative to strains without the sgRNAs. We found no genes that were consistently and significantly up-regulated, suggesting high specificity for each of the sgRNAs. Interestingly, we found a strikingly similar set of genes whose expression was significantly down-regulated in each of the individual sgRNA experiments (Figure 3A–D). Further examination revealed that all of these genes function in glucuronide utilization in *E. coli* (Figure 3E). Taken together, the data in Figures 2 and 3 establish that dCas9 when co-expressed with single sgRNAs can effectively and specifically down-regulate *gus* operon expression, leading to a regulatory cascade that represses expression of glucuronide utilization genes in *E. coli*.

### Inclusion of GusR on pGreg-dCas9 provides additive regulation

**Figure 4.**
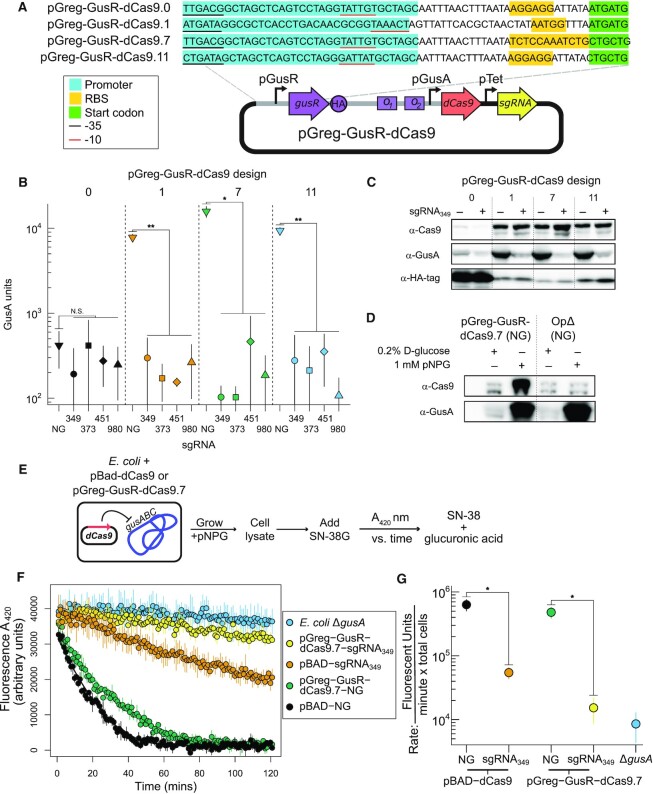
GusR-regulated dCas9 expression. (**A**) Top, schematic of regulatory regions of the pGreg-GusR-dCas9 constructs. pGreg-GusR-dCas9.0 represents the original construct. Highlighted are the sequences of the regulatory regions of each construct, including −35 and −10 promoter elements, ribosome-binding sites (RBS), and GusR start codons. At botton is a schematic of the plasmid (not to scale) with right-facing rectangles representing genes and right-facing arrows representing promoters. The rectangles represent GusR *O_1_* and *O_2_* sites upstream of the pGusA promoter that regulates dCas9 expression. (**B**) GusA activity assays with different pGreg-GusR-dCas9 constructs without an sgRNA (NG) or with one of four different sgRNAs (349, 373, 461, 980). (**C**) Western blots of *E. coli* cell extracts with indicated antibody on the left. The different lanes indicate plasmids coexpressing (+) or not coexpressing (−) sgRNA_349_. (**D**) Western blot of dCas9 and GusA expression in *E. coli* with the parental pGreg-GusR-dCas9.7 plasmid or the OpΔ plasmid grown in media supplemented with either glucose or pNPG. (**E**) Schematic for measuring SN-38G processing by *E. coli* cell extracts harbouring the indicated plasmids. (**F**) Plot of SN-38G utilization by *E. coli* cells extracts with the indicated plasmids. The plot is time (mins) versus fluorescence at 420 nm. (**G**) Rate of SN-38G hydrolysis for the indicated cell extracts calculated using the data shown in panel (E). Uncropped images for gel images in panels (C) and (D) are in Supplementary Figures S9 and S10, respectively. For panels (B), (F) and (G), data points are the mean of three biological replicates with the whiskers indicating the mean plus or minus the standard deviation. N.S. not significant, *P < 0.05, **P < 0.005 calculated by Welch’s *t*-test.

Encouraged by our findings that glucuronides can positively regulate expression of dCas9, we sought to improve on the pGreg-dCas9 design by including the GusR transcriptional repressor on the dCas9 plasmid, creating pGreg-GusR-dCas9 (Figure 4A, Supplementary Figure S1C). We rationalized that inclusion of GusR would provide stoichiometric regulation of dCas9 on the multi-copy plasmid, as opposed to the situation with pGreg-dCas9 where regulation relies on the single chromosomal copy of GusR. Moreover, the pGreg-GusR-dCas9 plasmid should be portable to different GUS-positive species where the chromosomal GusR may not recognize the *O_1_* and *O_2_* sequences derived from the *E. coli gusA* regulatory region. A C-terminal hemagglutinin (HA) tag was added to the *gusR* coding region to facilitate detection of GusR protein by western blots using an anti-HA antibody.

Our original pGreg-GusR-dCas9.0 construct used a constitutive Anderson promoter (BBa_J23118) to drive GusR expression. With this construct, and in the absence of a co-expressed sgRNA, we observed very low levels of GusA activity in *E. coli* cell extracts (Figure 4B, black inverted triangle). This low activity was mirrored by low levels of GusA protein observed with an anti-GusA antibody (Figure 4C, lane O). Moreover, we found very low levels of dCas9 protein, but very high levels of GusR protein (Figure 4C). We reasoned that the constitutive promoter driving GusR expression resulted in levels of GusR protein unresponsive to 1 mM pNPG or MetGluc. GusR would thus effectively act as a constitutive repressor of the *gus* operon and dCas9 expression. Adding a higher amount of pNPG (2−5 mM) resulted in slow growth of strains and did not relieve repression of GusA or dCas9.

We swapped out the regulatory region upstream of GusR with different combinations of promoter elements, mutated ribosome-binding sites, and non-AUG start codons to reduce GusR expression and increase dCas9 expression (Supplementary Figure S8A). We performed a screen to assess GusA activity in *E. coli* harbouring the different designs and found a range of activities for three independent clones of each design (Supplementary Figure S8B). From the different designs, we selected three sequence verified constructs (1, 7 and 11; Figure 4A) and showed that these designs had higher levels of GusA activity under inducing conditions (Figure 4B, orange, green and blue inverted triangles) as compared to the original construct (Figure 4B, black inverted triangle). The re-designed pGreg plasmids also showed increased amounts of dCas9 protein by western blot as compared to the original construct, regardless of whether an sgRNA was coexpressed (+ lanes) or not coexpressed (− lanes). GusR protein levels were also reduced compared to the original construct (Figure 4C, compare designs 1, 7 and 11 with 0). We individually cloned 4 sgRNAs (349, 373, 451, and 980) into the re-designed plasmids, and tested repression of GusA activity and expression under inducing conditions. As shown in Figure 4B, all three re-designed plasmids repressed GusA activity, regardless of the sgRNA used. GusA protein levels were also reduced when sgRNA_349_ was co-expressed (Figure 4C, compare − and + lanes with anti-GusA). We selected design 7 for further testing because it showed the highest GusA activity levels in the absence of sgRNAs, the lowest amount of GusR protein by western blot, and robust suppression of GusA activity when sgRNAs were co-expressed.

To provide further evidence that GusR is directly responsible for regulating dCas9 expression, we deleted the high affinity *O_1_* GusR operator site (44) from pGreg-GusR-dCas9.7, calling this plasmid OpΔ. We examined dCas9 expression by western blot with an anti-Cas9 antibody under repressive (glucose) or inducing (pNPG) culturing conditions with both the parental pGreg-GusR-dCas9.7 and OpΔ plasmids (Figure 4D, α-Cas9). We found little difference between dCas9 levels for the OpΔ plasmid in repressive or inducing conditions. This result contrasted with the strong increase in dCas9 expression with pGreg-GusR-dCas9.7 when pNPG was added to the media. There was no impact on GusA expression levels with either construct (Figure 4D, α-GusA). This result indicates that regulation of dCas9 expression from the OpΔ plasmid has been decoupled from GusR due to the deletion of the *O_1_* site, and is unresponsive to the addition of the pNPG inducer.

We next examined pGreg-GusR-dCas9.7 repression of GusA utilization of the glucuronidated form of irinotecan (SN-38G) using cell extracts of *E. coli* harbouring different dCas9 constructs (Figure 4E). SN-38G is a substrate for *E. coli* GusA, but *E. coli* cannot utilize SN-38G as a carbon source presumably because it lacks the appropriate transporter. In this assay, GusA utilization of SN-38G results in a decrease of fluorescence at 420 nm over time (Figure 4F, black dots) and a high rate of activity (Figure 4G, black dots, pBAD-dCas9 NG). Interestingly, we observed a reduction in SN-38G hydrolysis with the pGreg-GusR-dCas9.7 no sgRNA strain (Figure 4F, green dots) as compared to the pBAD-dCas9 NG experiment (Figure 4F, black dots), evidenced by the shallower slope of the fluorescent curve over time. This difference could be due to more stringent repression of *gusA* transcription by GusR expressed from the multi-copy pGreg plasmid than in the pBAD-dCas9 condition, where only the chromosomal copy of *gusR* is present. When an sgRNA (349) was cloned onto pGreg-GusR-dCas9.7 (Figure 4F and G, yellow dots), we observed further reduction of GusA hydrolysis of SN-38G to levels observed with an *E. coli* Δ*gusA* strain (Figure 4F and 4G, blue dots).

Taken together, these data show that addition of GusR to pGreg-dCas9 provides an additive effect to that of dCas9 for repression of GusA activity. In particular, pGreg-dCas9 can suppress GusA utilization of SN-38G compound to background levels similar to those observed with an *E. coli* Δ*gusA* strain. However, GusR expression levels must be tuned to balance responsiveness to exogenous glucuronide and dCas9 expression.

### Glucuronide-regulated dCas9 expression is restricted to GUS-positive bacteria

**Figure 5.**
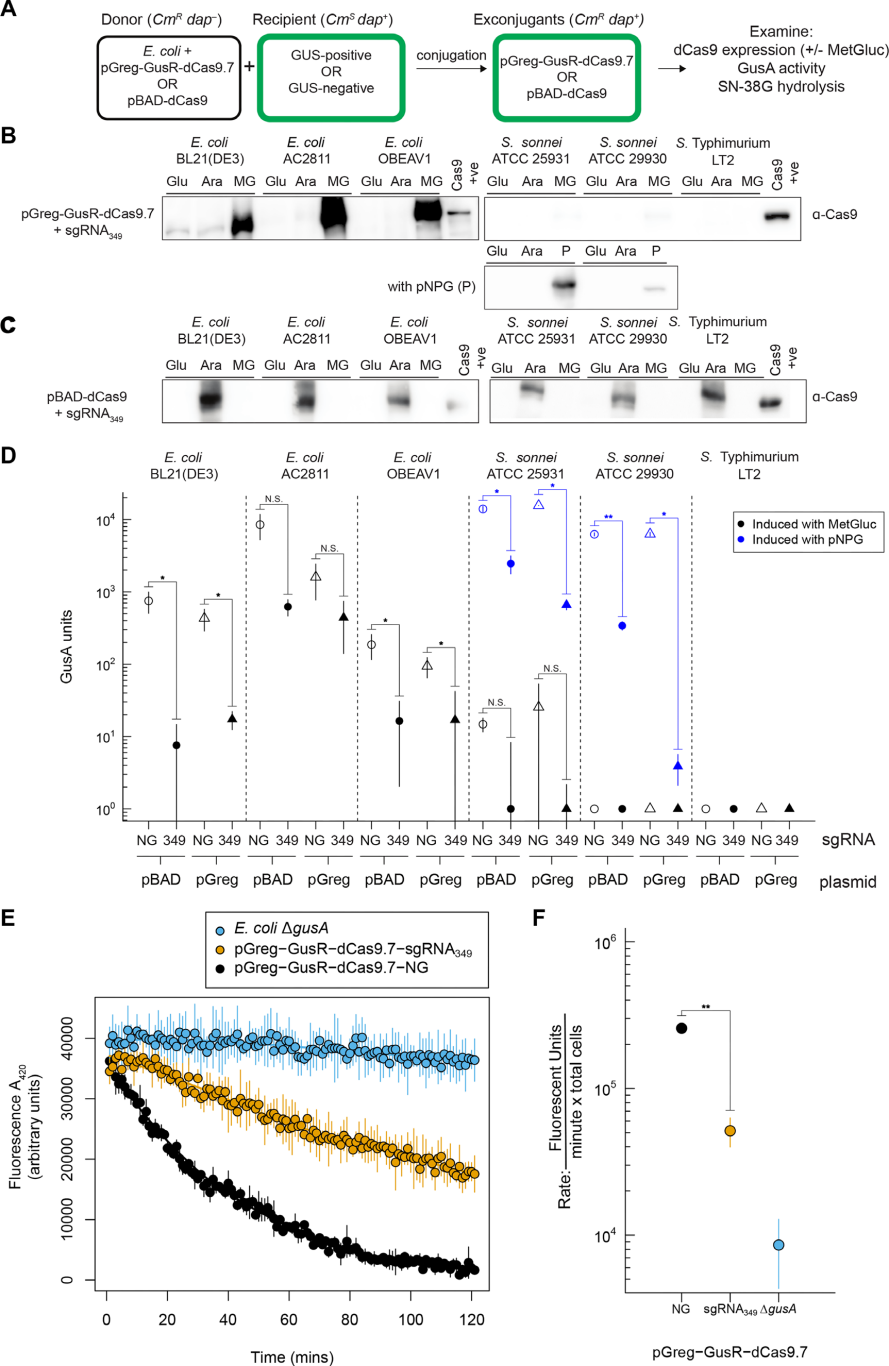
dCas9 expression with pGreg-GusR-dCas9.7 is limited to GUS-positive bacteria. (**A**) Strategy to examine dCas9 expression in GUS-positive or GUS-negative bacteria by conjugation of pGreg-GusR-dCas9. Cm^R^, chloramphenicol resistance. The *dap*^-^ donor strain has a knockout of the *dapA* gene that is used for counter selection during conjugation. (**B**) Western blots of dCas9 expression in exconjugants with pGreg-GusR-dCas9.7 in the indicated strains grown in liquid media supplemented with 0.2% d-glucose (Glu), 0.2% l-arabinose (Ara) or 1 mM MetGluc (MG). The Cas9+ lane is 5 ng of purified Cas9. (**C**) As for panel (B) but with exconjugants harbouring pBAD-dCas9. (**D**) GusA activity assays with the indicated strains harbouring either pBAD-dCas9 (pBAD) or pGreg-GusR-dCas9.7 (pGreg) without (NG) or with sgRNA_349_. Open and filled circles represent data for pBAD, and open and filled triangles represent data for pGreg. (**E**) SN-38G processing by *E. coli* OBEAV1 cell extracts harbouring pGreg-GusR-dCas9 with (orange circles) or without (black circles) sgRNA_349_. SN-38G hydrolysis in the *E. coli* Δ*gusA* strain is plotted for comparison. (**F**) Plot of SN-38G hydrolysis for the indicated cell extracts calculated using the data shown in panel (D). For panels (D), (E) and (F), data points are the mean of three biological replicates with the whiskers indicating the mean plus or minus the standard deviation. N.S. not significant, *P < 0.05, **P < 0.005 calculated by Welch’s *t*-test. Uncropped gel images in panels (B) and (C) are in Supplementary Figures S11 and S12, respectively, and uncropped gel images of the native microbiome species (to ensure no cross-reactivity occurred with the anti-Cas9 antibody) are in Supplementary Figure S13.

We next examined both pGreg-GusR-dCas9 and pBAD-dCas9 expression in different GUS-positive and GUS-negative enteric bacteria (Figure 5A). The rationale behind these experiments is outlined in Figure 1B and C, where dCas9 expressed from pGreg should be restricted to bacteria that are GUS-positive. In contrast, pBAD-dCas9 should be expressed by the addition of arabinose regardless of the GUS status of the bacteria. To test this hypothesis, we conjugated pGreg-GusR-dCas9.7+sgRNA_349_ from *E. coli* to GUS-positive (*E. coli* isolate AC2811 and OBEAV1, *S. sonnei* ATCC 25931) and GUS-negative (*S*. Typhimurium LT2, *S. sonnei* ATCC 29930) bacteria. *E. coli* BL21(DE3), a GUS-positive bacteria, served as a control for glucuronide-regulated dCas9 expression. dCas9 expression was examined by western blots under inducing conditions (1 mM MetGluc, MG), repressive conditions (0.2% d-glucose, Glu), and with 0.2% l-arabinose (Ara) (Figure 5B). We only observed dCas9 expression in GUS-positive bacteria and only when 1 mM MetGluc was added to the media (Figure 5B). The low *S. sonnei* GusA activity with MetGluc induction could reflect inefficient transport by *S. sonnei*, and explain why dCas9 induction was not visible by western blot in *S. sonnei*. When exconjugants of both *S. sonnei* ATCC 25931 and *S. sonnei* ATCC 29930 were grown with 1 mM pNPG induction instead of MetGluc, we found detectable levels of dCas9 expression by western blots (Figure 5B, with pNPG (P)). We also performed GusA activity assays and found much higher GusA activity from both *S. sonnei* species when they were induced with pNPG (Figure 5D, blue data points). GusA activity was lower with the presence of sgRNA_349_. In contrast, dCas9 expression was observed in all exconjugants with pBAD-dCas9 when 0.2% l-arabinose was added to media, regardless of whether the bacteria was GUS-positive or GUS-negative (Figure 5C).

We also examined GusA activity in exconjugants with pGreg-GusR-dCas9.7 or pBAD-dCas9. When sgRNA_349_ was present, we observed knockdown of GusA activity as compared to plasmids with no sgRNA (NG). Interestingly, GusA activity in exconjugants with pGreg-GusR-dCas9.7 and no sgRNA was lower than for the corresponding pBAD-dCas9 exconjugants of the two *E. coli* isolates (Figure 5D, open triangles versus open circles). This difference could be due to the added repressive effect of the plasmid-born copy of GusR on *gusA* transcription. A similar knockdown of GusA hydrolysis of SN-38G in cell extracts of *E. coli* OBEAV1 harbouring pGreg-GusR-dCas9.7-sgRNA_349_ was observed as compared to pGreg-GusR-dCas9.7-NG (Figure 5E and F).

### Repression of GusA activity in a mixed community

**Figure 6.**
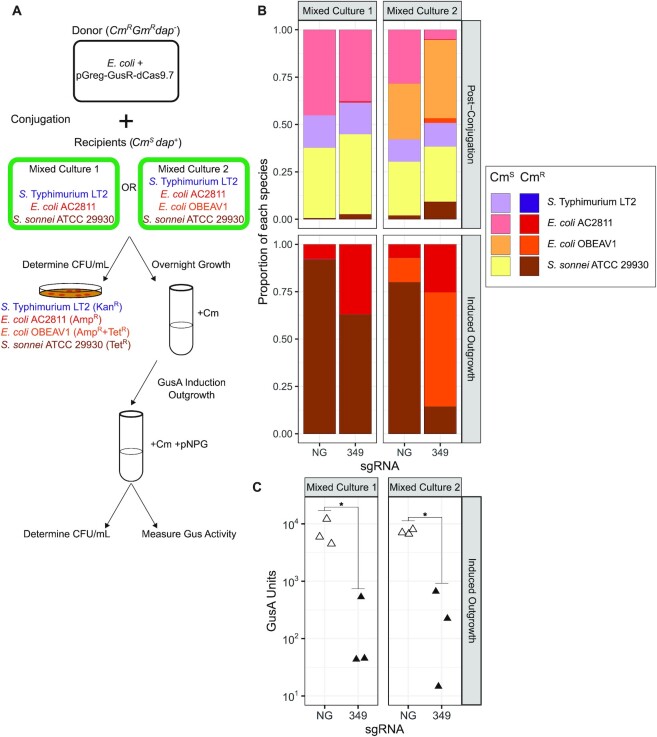
Repression of GusA in mixed cultures. (**A**) Strategy to examine repression of GusA in mixed cultures of bacteria by conjugation of pGreg-GusR-dCas9.7. Cm^R^, chloramphenicol resistance; Gm^R^, gentamycin resistance; Kan^R^, kanamycin resistance; Amp^R^, ampicillin resistance; Tet^R^, tetracycline resistance. (**B**) Mean proportions from three biological replicates of each species in the mixed cultures calculated from CFU/ml following conjugation of pGreg-GusR-dCas9.7 (pGreg) without (NG) or with sgRNA_349_. Aliquots of cultures post-conjugation (top panels) and after the induced outgrowth with pNPG (bottom panels) were spot-plated on the indicated resistance plates with and without chloramphenicol to count total recipients and exconjugants, respectively, of each species. Cultures were also plated with gentamycin and DAP to confirm donors had not survived. (**C**) GusA activity units from mixed cultures following conjugation of pGreg-GusR-dCas9.7 (pGreg) without (NG, open triangles) or with sgRNA_349_ (filled triangles). Data points from three biological replicates are shown. *P < 0.5 calculated by Welch's *t*-test.

To determine whether pGreg-GusR-dCas9.7 could repress GusA activity in a mixed bacterial community, we combined the *E. coli* ECGE101 donor strains harbouring pGreg-GusR-dCas9.7 (without (NG) or with sgRNA_349_) with three (*E. coli* AC2811, *S. sonnei* ATCC 29930, and *S*. Typhimurium LT2) or four (*E. coli* AC2811, *E. coli* OBEAV1, *S. sonnei* ATCC 29930 and *S*. Typhimurium LT2) recipient strains (Figure 6A), or with each species as a single recipient (Supplementary Figure 14). We measured the abundance of each strain after conjugation by selective plating on antibiotic-containing media, and observed conjugation frequencies of 0.009%-21% (Figure 6B). The mixed cultures from conjugation plates were grown overnight in selective media to eliminate any remaining *dapA^-^E. coli* donor, and then diluted into fresh media with pPNG to induce GusA expression. We measured the proportions of different strains that would contribute GusA activity (Figure 6B, bottom panels), finding that only Cm^R^*E. coli* AC2811, *E. coli* OBEAV1, and *S. sonnei* ATCC 29930 were present after the inducing outgrowth, and that *S*. Typhimurium LT2 was significantly outcompeted by the other strains. Interestingly, *S. sonnei* ATCC 29930 was outcompeted by both *E. coli* strains when sgRNA_349_ was present. This growth difference could be due to utilization of pNPG as a carbon source by the two *E. coli* species, as we noted less efficient knockdown of GusA in both *E. coli* species relative to knockdown in *S. sonnei* when we performed experiments with single species (Supplementary Figure 14). GusA activity of the mixed exconjugant communities was high (∼1000 units) with pGreg-GusR-dCas9.7 with no guide (NG) (Figure 6C, open triangles). In contrast, when sgRNA_349_ was present, GusA activity was lower by at least 10-fold for both mixed cultures. A similar knockdown of GusA activity was observed in mixed communities when experiments were performed with pBAD-dCas9 (Supplementary Figure 14), although the extent of GusA knockdown was lower than for pGreg-GusR-dCas9.7. Collectively, this data shows that pGreg-GusR-dCas9.7 or pBAD-dCas9 can be conjugated from *E. coli* to multiple Pseudomonadota in a mixed community to repress GusA activity.

## DISCUSSION

The ability to selectively regulate gene expression in bacteria containing the genetic machinery for metabolically relevant pathways within a microbial community can provide greater insight into the role of bacterial metabolism in human health and disease, and in other biological settings. The ease of targeting dCas9 to different sequences by simply changing the sgRNA sequence makes it an attractive tool to modulate metabolic activity. Indeed, dCas9 has been used to control gene expression in microbiomes by strategies such as species-specific promoters or inducible promoter systems that regulate dCas9 expression. Yet, these strategies cannot discriminate between bacteria with different metabolic capabilities simply because they provide no mechanism to limit dCas9 expression only to bacteria with a specific metabolic capacity. Linking dCas9 activity to the presence of a metabolic pathway provides more precise control of microbial metabolism, and potentially limits dCas9 off-target effects and the emergence of dCas9 inactivating mutations.

In this proof-of-concept study, we took advantage of the variable distribution of GUS enzymes in bacteria to genetically link dCas9 expression to the presence of the GUS pathway by re-purposing the GusR glucuronide-responsive transcription factor. A recent study suggested that over 100 xenobiotic medications may be substrates for GUS enzymes (46), emphasizing the importance of targeted regulation of GUS activity. We chose to re-purpose GusR, rather than the ExuR or UxuR transcription factors that function in the same pathway (65–68) to control dCas9 activity because GusR regulates expression of enzymes involved in the first step of glucuronide metabolism. Indeed, we found that dCas9 knockdown of *gusA* expression and activity was very effective in *E. coli*. Surprisingly, sgRNAs specific to *gusA* also resulted in the repression of multiple genes that are downstream of *gusA* in the glucuronide utilization pathway. It is possible that knockdown of *gusA* leads to downstream regulation through a cascade of transcriptional repression by the UxuR and ExuR proteins whose binding to regulatory regions is stimulated by the absence of D-glucuronate (Figure 3E) (65,68), which is the product of GusA hydrolysis of glucuronides (including pNPG).

Our data shows that plasmids with the GusR regulator (the pGreg-GusR-dCas9 series) provide an additional dCas9-independent repressive effect on GusA activity due to high GusR protein levels. This is most evident with the pGreg-GusR-dCas9.0 plasmid, where *E. coli* GusA activity is severely repressed in the absence of a sgRNA. The current pGreg plasmids use the GusR protein from *E. coli*, and thus would function most effectively in species where the *E. coli* GusR protein recognizes the chromosomal operator sites upstream of GusA. GusR homologs from other bacteria could be added to the dCas9 plasmid, or the *E. coli* GusR protein could be replaced, depending on the range of bacterial species that are intended targets of down-regulation. Genomic surveys of other GUS utilizing bacteria in the human gut revealed that GusR distribution is variable (44,65), suggesting that regulation of GusA activity is through a different (uncharacterized) transcription factor, or by a different mechanism altogether. In these bacteria, dCas9 activity could still be linked to glucuronide utilization by regulating its expression through transcription factors that function in the GUS pathway, such as UxuR or ExuR.

Bacteria possess a multitude of ligand-responsive transcription factors that respond to the intra- and extra-cellular environment to regulate gene expression. Many of these systems are uncharacterized (69), while others regulate metabolic processes relevant for human health and disease, such as trimethylamine (70) or *p*-cresol sulfonate metabolism (71). The strategy we outlined here could easily be applied to repurpose other ligand-responsive factors as biosensors to control expression of dCas9, or other genetic cargo, to regulate metabolic pathways. From a synthetic biology and biotechnology application perspective, ligand-responsive transcription factors have many uses as biosensors for controlling gene expression (72–74). The *gus* system provides an inducible expression tool that is complementary to described systems (75). Moreover, the fact that the *gus* system requires an appropriate glucuronide inducer and transporter (such as GusB) suggests regulatory strategies that could take advantage of the genetic background of the host bacteria. For instance, our experiments relied on the chromosomal copy of GusB, but GusB could be installed on the dCas9 expression plasmid to enable glucuronide regulation in both GUS-positive and -negative strains. Moreover, expression of dCas9 (or other cargo) can be further regulated by tuning the expression levels of GusR and hence the responsiveness to glucuronide.

## CONCLUSION

In summary, our results inform a general strategy to specifically link dCas9 expression to metabolic activities in relevant bacteria by re-purposing ligand-responsive transcription factors as biosensors. Our conjugative-plasmid delivery strategy does not require engineering of synthetic circuits in bacterial genomes, and could be applicable in a wide range of microbial communities.

## DATA AVAILABILITY

The RNAseq dataset generated in this study has been deposited in the Short Read Archive with the accession code PRJNA862923.

## Supplementary Material

gkac1248_Supplemental_Files

## References

[1] Vigouroux A. , Bikard D. CRISPR tools to control gene expression in bacteria. Microbiol. Mol. Biol. Rev. 2020; 84:e00077-19. 32238445 10.1128/MMBR.00077-19 PMC7117552

[2] Adli M. The CRISPR tool kit for genome editing and beyond. Nat. Commun. 2018; 9:1911. 29765029 10.1038/s41467-018-04252-2 PMC5953931

[3] Knott G.J. , Doudna J.A. CRISPR-Cas guides the future of genetic engineering. Science. 2018; 361:866–869. 30166482 10.1126/science.aat5011 PMC6455913

[4] Hamilton T.A. , Pellegrino G.M., Therrien J.A., Ham D.T., Bartlett P.C., Karas B.J., Gloor G.B., Edgell D.R. Efficient inter-species conjugative transfer of a CRISPR nuclease for targeted bacterial killing. Nat. Commun. 2019; 10:4544. 31586051 10.1038/s41467-019-12448-3 PMC6778077

[5] Citorik R.J. , Mimee M., Lu T.K. Sequence-specific antimicrobials using efficiently delivered RNA-guided nucleases. Nat. Biotech. 2014; 32:1141–1145. 10.1038/nbt.3011 PMC4237163 25240928

[6] Bikard D. , Euler C.W., Jiang W., Nussenzweig P.M., Goldberg G.W., Duportet X., Fischetti V.A., Marraffini L.A. Exploiting CRISPR-Cas nucleases to produce sequence-specific antimicrobials. Nat. Biotech. 2014; 32:1146–1150. 10.1038/nbt.3043 PMC4317352 25282355

[7] Gomaa A.A. , Klumpe H.E., Luo M.L., Selle K., Barrangou R., Beisel C.L. Programmable removal of bacterial strains by use of genome-targeting CRISPR-Cas systems. MBio. 2014; 5:e00928-13. 24473129 10.1128/mBio.00928-13 PMC3903277

[8] Neil K. , Allard N., Roy P., Grenier F., Menendez A., Burrus V., Rodrigue S. High-efficiency delivery of CRISPR-Cas9 by engineered probiotics enables precise microbiome editing. Mol. Syst. Biol. 2021; 17:e10335. 34665940 10.15252/msb.202110335 PMC8527022

[9] Lam K.N. , Spanogiannopoulos P., Soto-Perez P., Alexander M., Nalley M.J., Bisanz J.E., Nayak R.R., Weakley A.M., Feiqiao B.Y., Turnbaugh P.J. Phage-delivered CRISPR-Cas9 for strain-specific depletion and genomic deletions in the gut microbiome. Cell Rep. 2021; 37:109930. 34731631 10.1016/j.celrep.2021.109930 PMC8591988

[10] Qi L.S. , Larson M.H., Gilbert L.A., Doudna J.A., Weissman J.S., Arkin A.P., Lim W.A. Repurposing CRISPR as an RNA-guided platform for sequence-specific control of gene expression. Cell. 2013; 152:1173–1183. 23452860 10.1016/j.cell.2013.02.022 PMC3664290

[11] Maurice C.F. , Haiser H.J., Turnbaugh P.J. Xenobiotics shape the physiology and gene expression of the active human gut microbiome. Cell. 2013; 152:39–50. 23332745 10.1016/j.cell.2012.10.052 PMC3552296

[12] Gevers D. , Kugathasan S., Denson L.A., Vázquez-Baeza Y., Van Treuren W., Ren B., Schwager E., Knights D., Song S.J., Yassour M. et al. The treatment-naive microbiome in new-onset Crohn’s disease. Cell Host Microbe. 2014; 15:382–392. 24629344 10.1016/j.chom.2014.02.005 PMC4059512

[13] Frank D.N. , Amand A. L.S., Feldman R.A., Boedeker E.C., Harpaz N., Pace N.R. Molecular-phylogenetic characterization of microbial community imbalances in human inflammatory bowel diseases. Proc. Natl. Acad. Sci. U.S.A. 2007; 104:13780–13785. 17699621 10.1073/pnas.0706625104 PMC1959459

[14] Jiang H. , Ling Z., Zhang Y., Mao H., Ma Z., Yin Y., Wang W., Tang W., Tan Z., Shi J. et al. Altered fecal microbiota composition in patients with major depressive disorder. Brain Behav. Immun. 2015; 48:186–194. 25882912 10.1016/j.bbi.2015.03.016

[15] Zheng P. , Zeng B., Zhou C., Liu M., Fang Z., Xu X., Zeng L., Chen J., Fan S., Du X. et al. Gut microbiome remodeling induces depressive-like behaviors through a pathway mediated by the host’s metabolism. Mol. Psychiatr. 2016; 21:786–796. 10.1038/mp.2016.44 27067014

[16] Kostic A.D. , Chun E., Robertson L., Glickman J.N., Gallini C.A., Michaud M., Clancy T.E., Chung D.C., Lochhead P., Hold G.L. et al. Fusobacterium nucleatum potentiates intestinal tumorigenesis and modulates the tumor-immune microenvironment. Cell Host Microbe. 2013; 14:207–215. 23954159 10.1016/j.chom.2013.07.007 PMC3772512

[17] Sun J. , Kato I. Gut microbiota, inflammation and colorectal cancer. Genes Dis. 2016; 3:130–143. 28078319 10.1016/j.gendis.2016.03.004 PMC5221561

[18] Yu Y. , Champer J., Beynet D., Kim J., Friedman A.J. The role of the cutaneous microbiome in skin cancer: lessons learned from the gut. J. Drugs Dermatol. 2015; 14:461–465. 25942663

[19] Tremaroli V. , Bäckhed F. Functional interactions between the gut microbiota and host metabolism. Nature. 2012; 489:242–249. 22972297 10.1038/nature11552

[20] Zimmermann M. , Zimmermann-Kogadeeva M., Wegmann R., Goodman A.L. Mapping human microbiome drug metabolism by gut bacteria and their genes. Nature. 2019; 570:462–467. 31158845 10.1038/s41586-019-1291-3 PMC6597290

[21] Gilbert J.A. , Quinn R.A., Debelius J., Xu Z.Z., Morton J., Garg N., Jansson J.K., Dorrestein P.C., Knight R. Microbiome-wide association studies link dynamic microbial consortia to disease. Nature. 2016; 535:94–103. 27383984 10.1038/nature18850

[22] Koenig J.E. , Spor A., Scalfone N., Fricker A.D., Stombaugh J., Knight R., Angenent L.T., Ley R.E. Succession of microbial consortia in the developing infant gut microbiome. Proc. Natl. Acad. Sci. U.S.A. 2011; 108:4578–4585. 20668239 10.1073/pnas.1000081107 PMC3063592

[23] Macklaim J.M. , Fernandes A.D., Di Bella J.M., Hammond J.-A., Reid G., Gloor G.B. Comparative meta-RNA-seq of the vaginal microbiota and differential expression by *Lactobacillus iners* in health and dysbiosis. Microbiome. 2013; 1:12. 24450540 10.1186/2049-2618-1-12 PMC3971606

[24] Cui L. , Vigouroux A., Rousset F., Varet H., Khanna V., Bikard D. A CRISPRi screen in *E. coli* reveals sequence-specific toxicity of dCas9. Nat. Commun. 2018; 9:1912. 29765036 10.1038/s41467-018-04209-5 PMC5954155

[25] Cho S. , Choe D., Lee E., Kim S.C., Palsson B., Cho B.-K. High-level dCas9 expression induces abnormal cell morphology in *Escherichia coli*. ACS Synthetic Biology. 2018; 7:1085–1094. 29544049 10.1021/acssynbio.7b00462

[26] Nielsen A.A. , Voigt C.A. Multi-input CRISPR/C as genetic circuits that interface host regulatory networks. Mol. Syst. Biol. 2014; 10:763. 25422271 10.15252/msb.20145735 PMC4299604

[27] Lim B. , Zimmermann M., Barry N.A., Goodman A.L. Engineered regulatory systems modulate gene expression of human commensals in the gut. Cell. 2017; 169:547–558. 28431252 10.1016/j.cell.2017.03.045 PMC5532740

[28] Taketani M. , Zhang J., Zhang S., Triassi A.J., Huang Y.-J., Griffith L.G., Voigt C.A. Genetic circuit design automation for the gut resident species *Bacteroides thetaiotaomicron*. Nat. Biotech. 2020; 38:962–969. 10.1038/s41587-020-0468-5 PMC8922546 32231334

[29] Mimee M. , Tucker A.C., Voigt C.A., Lu T.K. Programming a human commensal bacterium, *Bacteroides thetaiotaomicron*, to sense and respond to stimuli in the murine gut microbiota. Cell Syst. 2015; 1:62–71. 26918244 10.1016/j.cels.2015.06.001 PMC4762051

[30] Hsu B.B. , Plant I.N., Lyon L., Anastassacos F.M., Way J.C., Silver P.A. In situ reprogramming of gut bacteria by oral delivery. Nat. Commun. 2020; 11:5030. 33024097 10.1038/s41467-020-18614-2 PMC7538559

[31] Pellock S.J. , Redinbo M.R. Glucuronides in the gut: Sugar-driven symbioses between microbe and host. J. Biol. Chem. 2017; 292:8569–8576. 28389557 10.1074/jbc.R116.767434 PMC5448086

[32] Pollet R.M. , D’Agostino E.H., Walton W.G., Xu Y., Little M.S., Biernat K.A., Pellock S.J., Patterson L.M., Creekmore B.C., Isenberg H.N. et al. An atlas of β-glucuronidases in the human intestinal microbiome. Structure. 2017; 25:967–977. 28578872 10.1016/j.str.2017.05.003 PMC5533298

[33] Koropatkin N.M. , Cameron E.A., Martens E.C. How glycan metabolism shapes the human gut microbiota. Nat. Rev. Microbiol. 2012; 10:323–335. 22491358 10.1038/nrmicro2746 PMC4005082

[34] Oren A. , Garrity G.M. Valid publication of the names of forty-two phyla of prokaryotes. Int. J. Syst. Evol. Microbiol. 2021; 71:005056. 10.1099/ijsem.0.005056 34694987

[35] Blanco C. , Ritzenthaler P., Mata-Gilsinger M. Cloning and endonuclease restriction analysis of *uidA* and *uidR* genes in *Escherichia coli* K-12: determination of transcription direction for the *uidA* gene. J. Bacteriol. 1982; 149:587–594. 6276362 10.1128/jb.149.2.587-594.1982 PMC216546

[36] Liang W.-J. , Wilson K.J., Xie H., Knol J., Suzuki S., Rutherford N.G., Henderson P.J., Jefferson R.A. The *gusBC* genes of *Escherichia coli* encode a glucuronide transport system. J. Bacteriol. 2005; 187:2377–2385. 15774881 10.1128/JB.187.7.2377-2385.2005 PMC1065211

[37] Wilson K.J. , Hughes S.G., Jefferson R.A. The *Escherichia coli**gus* operon: induction and expression of the *gus* operon in *E. coli* and the occurrence and use of GUS in other bacteria. GUS protocols. Using the GUS gene as reporter of gene expression. 1992; Academic Press Inc 7–22.

[38] Blanco C. , Ritzenthaler P., Mata-Gilsinger M. Nucleotide sequence of a regulatory region of the *uidA* gene in *Escherichia coli* K12. Mol. Gen. Genet. MGG. 1985; 199:101–105. 3889544 10.1007/BF00327517

[39] Novel M. , Novel G. Regulation of beta-glucuronidase synthesis in *Escherichia coli* K-12: constitutive mutants specifically derepressed for uidA expression. J. Bacteriol. 1976; 127:406–417. 776933 10.1128/jb.127.1.406-417.1976 PMC233074

[40] Novel G. , Didier-Fichet M., Stoeber F. Inducibility of β-glucuronidase in wild-type and hexuronate-negative mutants of *Escherichia coli* K-12. J. Bacteriol. 1974; 120:89–95. 4607717 10.1128/jb.120.1.89-95.1974 PMC245734

[41] Ritzenthaler P. , Blanco C., Mata-Gilsinger M. Interchangeability of repressors for the control of the *uxu* and *uid* operons in *E. coli* K12. Mol. Gen. Genet. MGG. 1983; 191:263–270. 6353163 10.1007/BF00334824

[42] Ritzenthaler P. , Mata-Gilsinger M. Use of *in vitro* gene fusions to study the *uxuR* regulatory gene in *Escherichia coli* K-12: direction of transcription and regulation of its expression. J. Bacteriol. 1982; 150:1040–1047. 6281232 10.1128/jb.150.3.1040-1047.1982 PMC216320

[43] Ramos J.L. , Martínez-Bueno M., Molina-Henares A.J., Terán W., Watanabe K., Zhang X., Gallegos M.T., Brennan R., Tobes R. The TetR family of transcriptional repressors. Microbiol. Mol. Biol. Rev. 2005; 69:326–356. 15944459 10.1128/MMBR.69.2.326-356.2005 PMC1197418

[44] Little M.S. , Pellock S.J., Walton W.G., Tripathy A., Redinbo M.R. Structural basis for the regulation of β-glucuronidase expression by human gut Enterobacteriaceae. Proc. Natl. Acad. Sci. U.S.A. 2018; 115:E152–E161. 29269393 10.1073/pnas.1716241115 PMC5777068

[45] Novel G. , Novel M. Mutants *d’Escherichia coli* K 12 affectés pour leur croissance sur méthyl-β-D-glucuronide: Localisation du gène de structure de la β-D-glucuronidase (uid A). Mol. Gen. Genet. 1973; 120:319–335. 4568840

[46] Elmassry M.M. , Kim S., Busby B. Predicting drug-metagenome interactions: variation in the microbial β-glucuronidase level in the human gut metagenomes. PloS One. 2021; 16:e0244876. 33411719 10.1371/journal.pone.0244876 PMC7790408

[47] Jariwala P.B. , Pellock S.J., Goldfarb D., Cloer E.W., Artola M., Simpson J.B., Bhatt A.P., Walton W.G., Roberts L.R., Major M.B., et al. Discovering the microbial enzymes driving drug toxicity with activity-based protein profiling. ACS Chem. Biol. 2019; 15:217–225. 31774274 10.1021/acschembio.9b00788 PMC7321802

[48] Dashnyam P. , Mudududdla R., Hsieh T.-J., Lin T.-C., Lin H.-Y., Chen P.-Y., Hsu C.-Y., Lin C.-H. β-Glucuronidases of opportunistic bacteria are the major contributors to xenobiotic-induced toxicity in the gut. Sci. Rep. 2018; 8:16372. 30401818 10.1038/s41598-018-34678-z PMC6219552

[49] Bhatt A.P. , Pellock S.J., Biernat K.A., Walton W.G., Wallace B.D., Creekmore B.C., Letertre M.M., Swann J.R., Wilson I.D., Roques J.R. et al. Targeted inhibition of gut bacterial β-glucuronidase activity enhances anticancer drug efficacy. Proc. Natl. Acad. Sci. U.S.A. 2020; 117:7374–7381. 32170007 10.1073/pnas.1918095117 PMC7132129

[50] Wallace B.D. , Roberts A.B., Pollet R.M., Ingle J.D., Biernat K.A., Pellock S.J., Venkatesh M.K., Guthrie L., O’Neal S.K., Robinson S.J. et al. Structure and inhibition of microbiome β-glucuronidases essential to the alleviation of cancer drug toxicity. Chem. Biol. 2015; 22:1238–1249. 26364932 10.1016/j.chembiol.2015.08.005 PMC4575908

[51] Brumwell S.L. , MacLeod M.R., Huang T., Cochrane R.R., Meaney R.S., Zamani M., Matysiakiewicz O., Dan K.N., Janakirama P., Edgell D.R., et al. Designer *Sinorhizobium meliloti* strains and multi-functional vectors enable direct inter-kingdom DNA transfer. PLoS One. 2019; 14:e0206781. 31206509 10.1371/journal.pone.0206781 PMC6576745

[52] Slattery S.S. , Diamond A., Wang H., Therrien J.A., Lant J.T., Jazey T., Lee K., Klassen Z., Desgagne-Penix I., Karas B.J. et al. An expanded plasmid-based genetic toolbox enables Cas9 genome editing and stable maintenance of synthetic pathways in *Phaeodactylum tricornutum*. ACS Synt. Biol. 2018; 7:328–338. 10.1021/acssynbio.7b00191 29298053

[53] Guzman L.-M. , Belin D., Carson M.J., Beckwith J. Tight regulation, modulation, and high-level expression by vectors containing the arabinose PBAD promoter. J. Bacteriol. 1995; 177:4121–4130. 7608087 10.1128/jb.177.14.4121-4130.1995 PMC177145

[54] Wolfs J.M. , Hamilton T.A., Lant J.T., Laforet M., Zhang J., Salemi L.M., Gloor G.B., Schild-Poulter C., Edgell D.R. Biasing genome-editing events toward precise length deletions with an RNA-guided TevCas9 dual nuclease. Proc. Natl. Acad. Sci. U.S.A. 2016; 113:14988–14993. 27956611 10.1073/pnas.1616343114 PMC5206545

[55] Engler C. , Kandzia R., Marillonnet S. A one pot, one step, precision cloning method with high throughput capability. PLoS One. 2008; 3:e3647. 18985154 10.1371/journal.pone.0003647 PMC2574415

[56] Strand T.A. , Lale R., Degnes K.F., Lando M., Valla S. A new and improved host-independent plasmid system for RK2-based conjugal transfer. PLoS One. 2014; 9:e90372. 24595202 10.1371/journal.pone.0090372 PMC3940858

[57] Bolger A.M. , Lohse M., Usadel B. Trimmomatic: a flexible trimmer for Illumina sequence data. Bioinformatics. 2014; 30:2114–2120. 24695404 10.1093/bioinformatics/btu170 PMC4103590

[58] Kim D. , Langmead B., Salzberg S.L. HISAT: a fast spliced aligner with low memory requirements. Nat. Methods. 2015; 12:357–360. 25751142 10.1038/nmeth.3317 PMC4655817

[59] Putri G.H. , Anders S., Pyl P.T., Pimanda J.E., Zanini F. Analysing high-throughput sequencing data in Python with HTSeq 2.0. Bioinformatics. 2022; 38:2943–2945. 35561197 10.1093/bioinformatics/btac166 PMC9113351

[60] Love M.I. , Huber W., Anders S. Moderated estimation of fold change and dispersion for RNA-seq data with DESeq2. Genome Biol. 2014; 15:550. 25516281 10.1186/s13059-014-0550-8 PMC4302049

[61] Tryland I. , Fiksdal L. Enzyme characteristics of β-D-galactosidase-and β-D-glucuronidase-positive bacteria and their interference in rapid methods for detection of waterborne coliforms and *Escherichia coli*. Appl. Environ. Microbiol. 1998; 64:1018–1023. 9501441 10.1128/aem.64.3.1018-1023.1998 PMC106360

[62] Kim T. , Han J.-I. Fast detection and quantification of *Escherichia coli* using the base principle of the microbial fuel cell. J. Environ. Manage. 2013; 130:267–275. 24095789 10.1016/j.jenvman.2013.08.051

[63] Bikard D. , Jiang W., Samai P., Hochschild A., Zhang F., Marraffini L.A. Programmable repression and activation of bacterial gene expression using an engineered CRISPR-Cas system. Nucleic Acids Res. 2013; 41:7429–7437. 23761437 10.1093/nar/gkt520 PMC3753641

[64] Reis A.C. , Halper S.M., Vezeau G.E., Cetnar D.P., Hossain A., Clauer P.R., Salis H.M. Simultaneous repression of multiple bacterial genes using nonrepetitive extra-long sgRNA arrays. Nat. Biotech. 2019; 37:1294–1301. 10.1038/s41587-019-0286-9 31591552

[65] Suvorova I.A. , Tutukina M.N., Ravcheev D.A., Rodionov D.A., Ozoline O.N., Gelfand M.S. Comparative genomic analysis of the hexuronate metabolism genes and their regulation in gammaproteobacteria. J. Bacteriol. 2011; 193:3956–3963. 21622752 10.1128/JB.00277-11 PMC3147527

[66] Shimada T. , Ogasawara H., Ishihama A. Single-target regulators form a minor group of transcription factors in *Escherichia coli* K-12. Nucleic Acids Res. 2018; 46:3921–3936. 29529243 10.1093/nar/gky138 PMC5934670

[67] Ishihama A. , Shimada T., Yamazaki Y. Transcription profile of *Escherichia coli*: genomic SELEX search for regulatory targets of transcription factors. Nucleic Acids Res. 2016; 44:2058–2074. 26843427 10.1093/nar/gkw051 PMC4797297

[68] Tutukina M.N. , Potapova A.V., Cole J.A., Ozoline O.N. Control of hexuronate metabolism in *Escherichia coli* by the two interdependent regulators, ExuR and UxuR: derepression by heterodimer formation. Microbiology. 2016; 162:1220–1231. 27129867 10.1099/mic.0.000297

[69] Hanko E.K. , Paiva A.C., Jonczyk M., Abbott M., Minton N.P., Malys N. A genome-wide approach for identification and characterisation of metabolite-inducible systems. Nat. Commun. 2020; 11:1213. 32139676 10.1038/s41467-020-14941-6 PMC7057948

[70] Zeisel S.H. , Warrier M. Trimethylamine N-oxide, the microbiome, and heart and kidney disease. Ann. Rev. Nutr. 2017; 37:157–181. 28715991 10.1146/annurev-nutr-071816-064732

[71] Visconti A. , Le Roy C.I., Rosa F., Rossi N., Martin T.C., Mohney R.P., Li W., de Rinaldis E., Bell J.T., Venter J.C. et al. Interplay between the human gut microbiome and host metabolism. Nat. Commun. 2019; 10:4505. 31582752 10.1038/s41467-019-12476-z PMC6776654

[72] Wu Y. , Du G., Chen J., Liu L. Genetically encoded biosensors and their applications in the development of microbial cell factories. Engineering of Microbial Biosynthetic Pathways. 2020; Springer 53–73.

[73] Mahr R. , Frunzke J. Transcription factor-based biosensors in biotechnology: current state and future prospects. Appl. Microbiol. Biotechn. 2016; 100:79–90. 10.1007/s00253-015-7090-3 PMC4700088 26521244

[74] Mannan A.A. , Liu D., Zhang F., Oyarzún D.A. Fundamental design principles for transcription-factor-based metabolite biosensors. ACS Synt. Biol. 2017; 6:1851–1859. 10.1021/acssynbio.7b00172 28763198

[75] Rogers J.K. , Guzman C.D., Taylor N.D., Raman S., Anderson K., Church G.M. Synthetic biosensors for precise gene control and real-time monitoring of metabolites. Nucleic Acids Res. 2015; 43:7648–7660. 26152303 10.1093/nar/gkv616 PMC4551912

