# Supplementary material for: Metabolically-targeted dCas9 expression in bacteria
Source: Nucleic Acids Res. 2023 Jan 11;51(2):982–96. doi: 10.1093/nar/gkac1248 (PMC9881133; doi:10.1093/nar/gkac1248)
Supplement: gkac1248_Supplemental_Files [file gkac1248_supplemental_files.zip › Pellegrino_supplemental.pdf]

Supplementary Data for:
Metabolically-targeted dCas9 expression in bacteria

Gregory M. Pellegrino[1], Tyler S. Browne[1], Keerthana Sharath[1], Khaleda A. Bari[1], Sarah Vancuren[2], Emma Allen-Vercoe[2], Gregory B. Gloor[1], and David R. Edgell[1*]

[1]Schulich School of Medicine and Dentistry, Department of Biochemistry, London, Ontario, Canada
[2]Department of Molecular and Cellular Biology, University of Guelph, Guelph, Ontario, Canada
[*]correspondence to: dedgell@uwo.ca

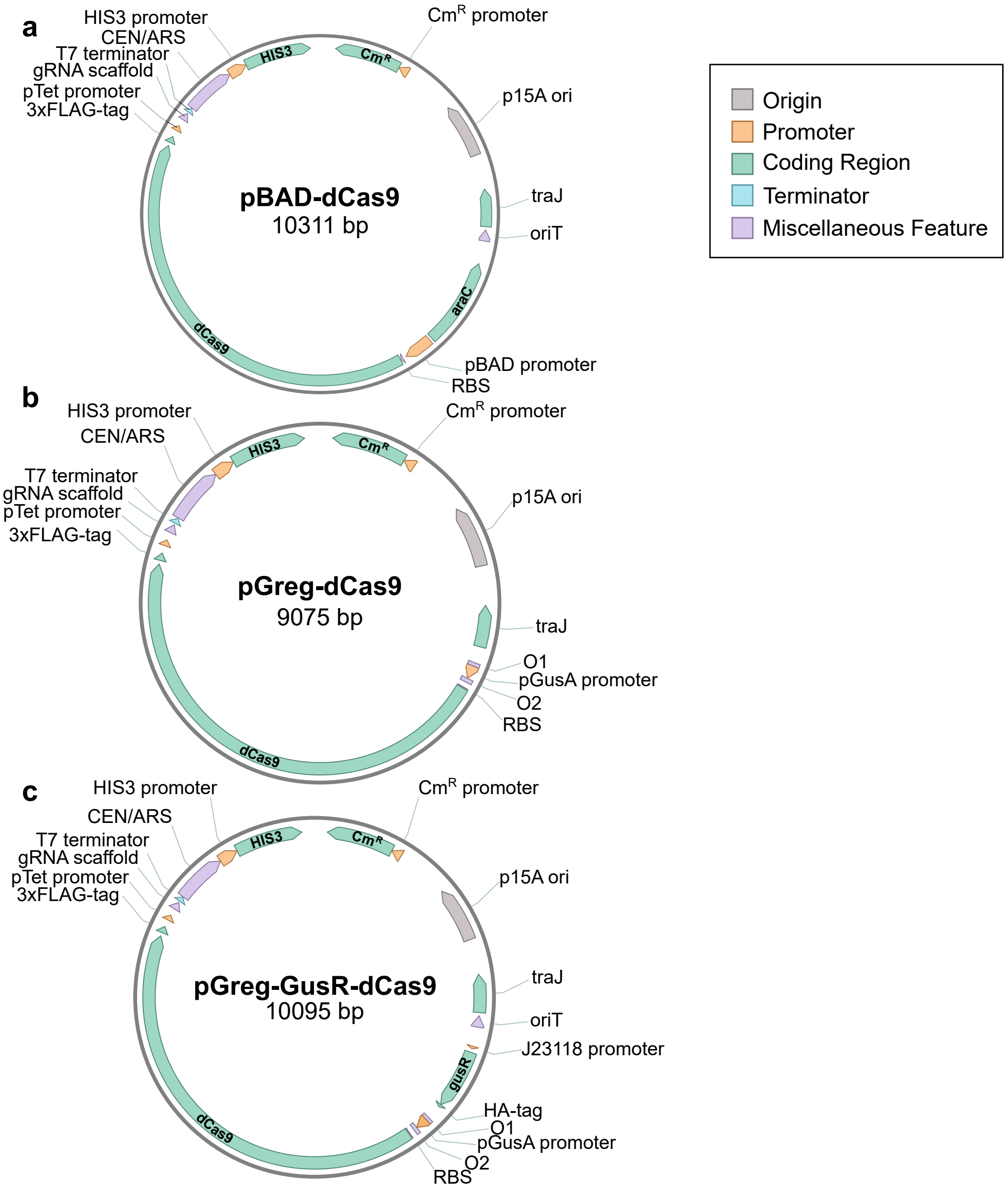

Figure S1: Detailed maps of plasmids introduced in this study. **a** The pBAD-dCas9 plasmid. CEN/ARS, *S. cerevisiae* CEN6 centromere fused to an autonomously replicating sequence; HIS3, imidazoleglycerol-phosphate dehydratase required for histidine biosynthesis, yeast auxotrophic marker; Cm^R, chloramphenicol acetyl-transferase resistance gene; p15A ori, medium-copy-number p15A origin of replication; traJ, oriT-recognizing protein; oriT, incP origin of transfer; araC, L-arabinose regulatory protein; pBAD promoter, promoter from the *E. coli* L-arabinose operon; dCas9, *S. pyogenes* Cas9, catalytically inactivated by D10A and H841A mutations, with a C-terminal 3X FLAG tag; pTet, promoter from the tetracycline resistance gene; gRNA scaffold, RNA scaffold sequence necessary for Cas-binding; T7 terminator, transcription terminator for bacteriophage T7 RNA polymerase. **b** The pGreg-dCas9 plasmid. O1 and O2, GusR operator binding sites; pGusA promoter, promoter from the gusA regulatory region; all other labels are as previously described in panel **a**. **c** The pGreg-GusR-dCas9 plasmid. J23118 promoter, constitutive Anderson promoter part BBa_J23118; gusR, repressor for the GUS operon from *E. coli* with a C-terminal HA-tag; all other labels are as previously described in panel **a**.

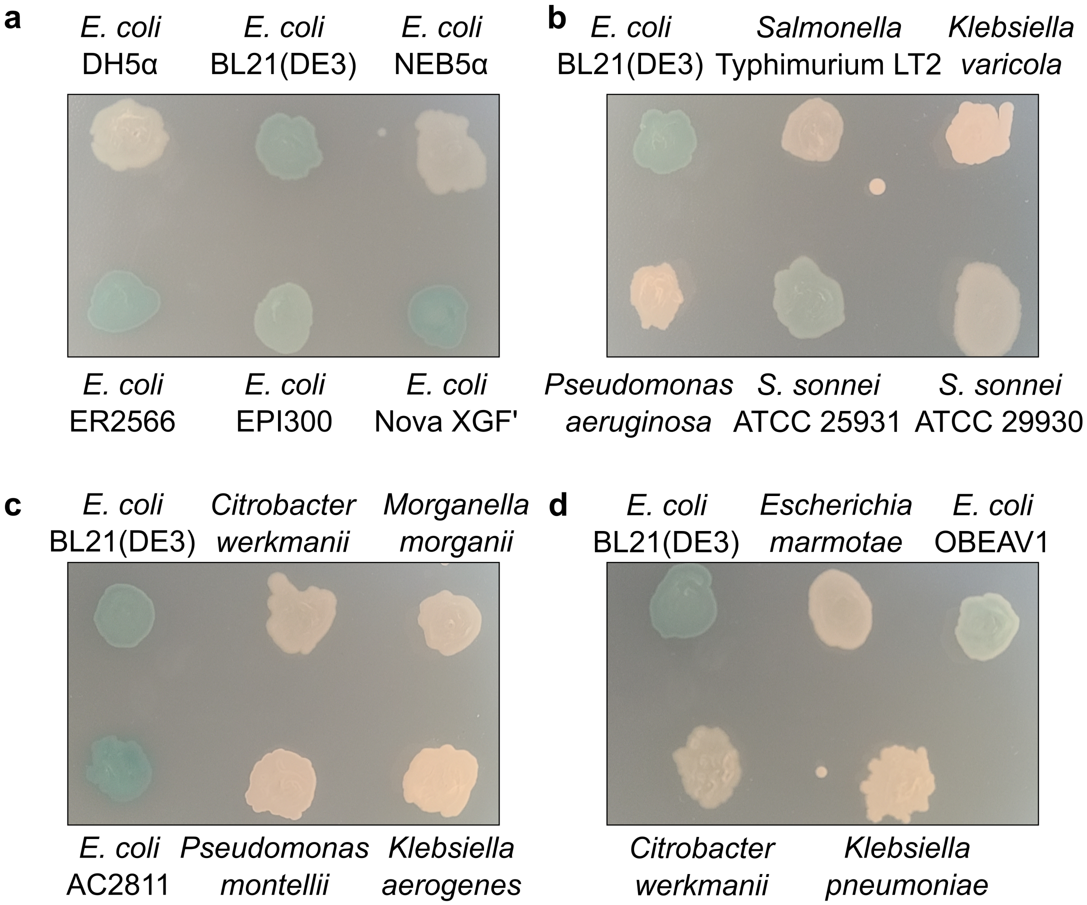

Figure S2: Blue-white GusA activity screen with X-Gluc (5-bromo-4-chloro-3-indolyl-β-D-glucuronic acid, cyclohexylammonium salt, 40 µg/mL in non-selective LB agar). **a** Six *E. coli* strains were spotted onto an LB X-Gluc plate to determine if they exhibited GusA activity. **b**-**d** Microbiome-derived bacterial species were spotted onto LB X-Gluc plates (with *E. coli* BL21(DE3) as a positive control) to determine which species expressed functional GusA.

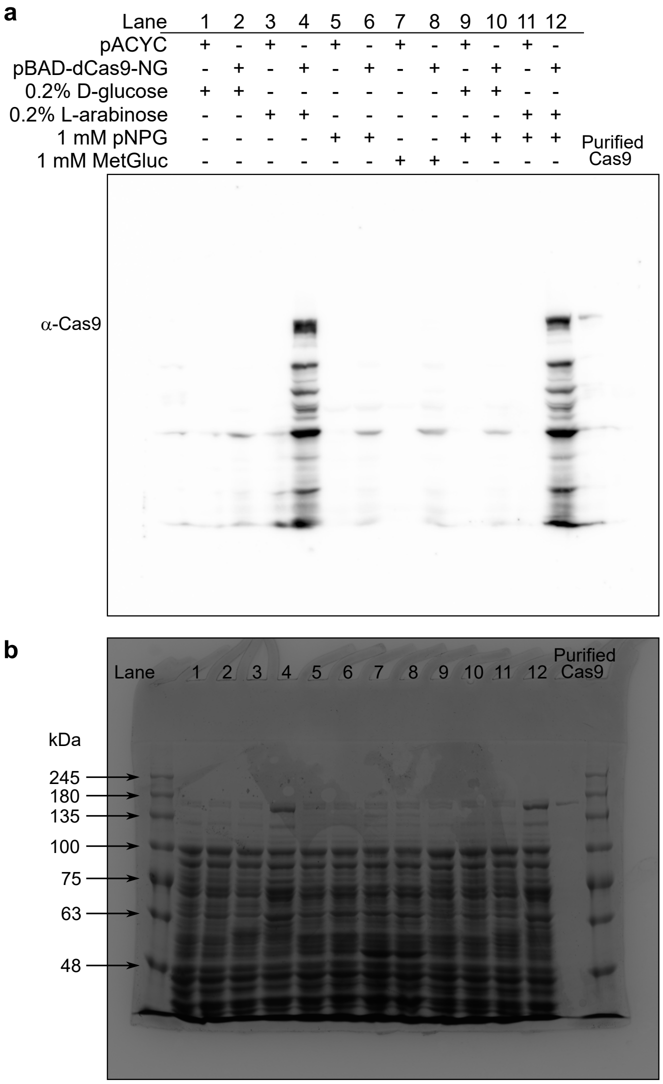

Figure S3: Raw images associated with figure 2C. **a** Expression of dCas9 under the indicated conditions with *E. coli* harbouring the pACYC backbone plasmid or pBAD-dCas9 assessed by western blots with a polyclonal anti-Cas9 antibody. **b** Raw image of identical protein samples stained with Coomassie blue.

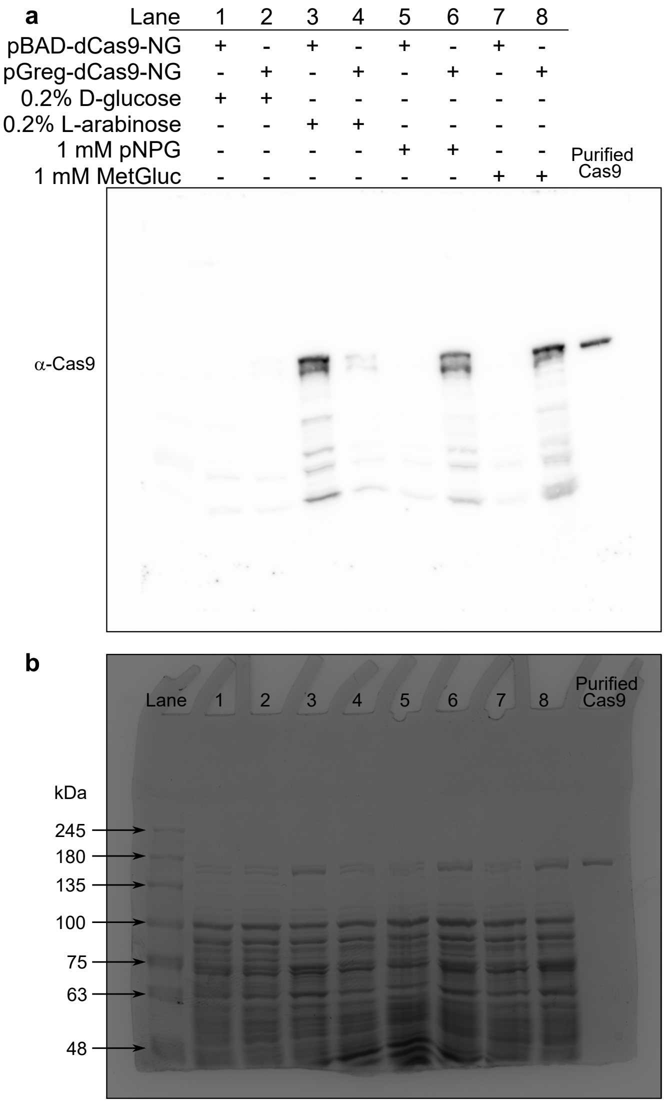

Figure S4: Raw images associated with figure 2D. **a** Expression of dCas9 under the indicated conditions with *E. coli* harbouring pBAD-dCas9 or pGreg-dCas9 assessed by western blots with a polyclonal anti-Cas9 antibody. **b** Raw image of identical protein samples stained with Coomassie blue.

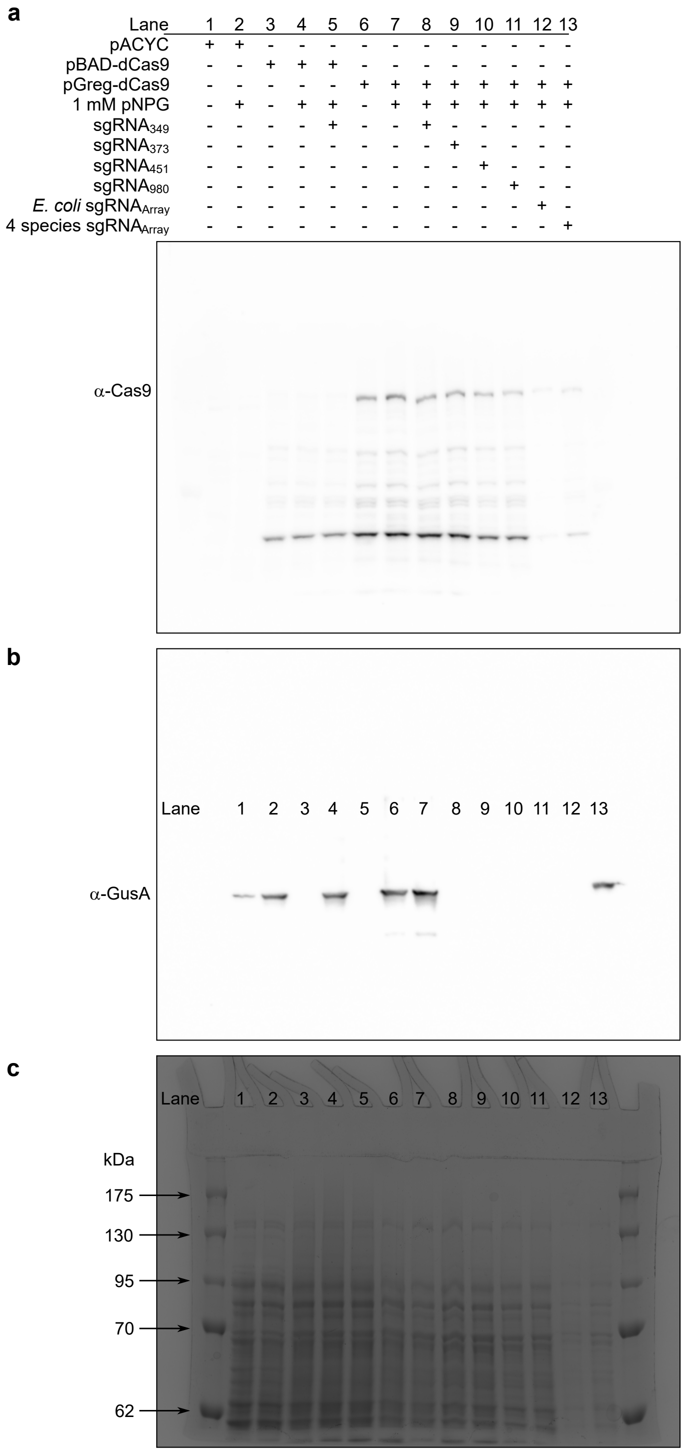

Figure S5: Raw images associated with figure 2h. Expression of **a** dCas9 and **b** GusA under the indicated conditions with *E. coli* harbouring the pACYC backbone plasmid, pBAD-dCas9 with and without $sgRNA_{349}$, or pGreg-dCas9 with and without the indicated sgRNAs assessed by western blots with a polyclonal anti-Cas9 antibody and a polyclonal anti-GusA antibody, respectively. The 4 species $sgRNA_{Array}$ was an alternate design of an sgRNA array that was not examined further in the main paper body. **c** Raw image of identical protein samples stained with Coomassie blue.

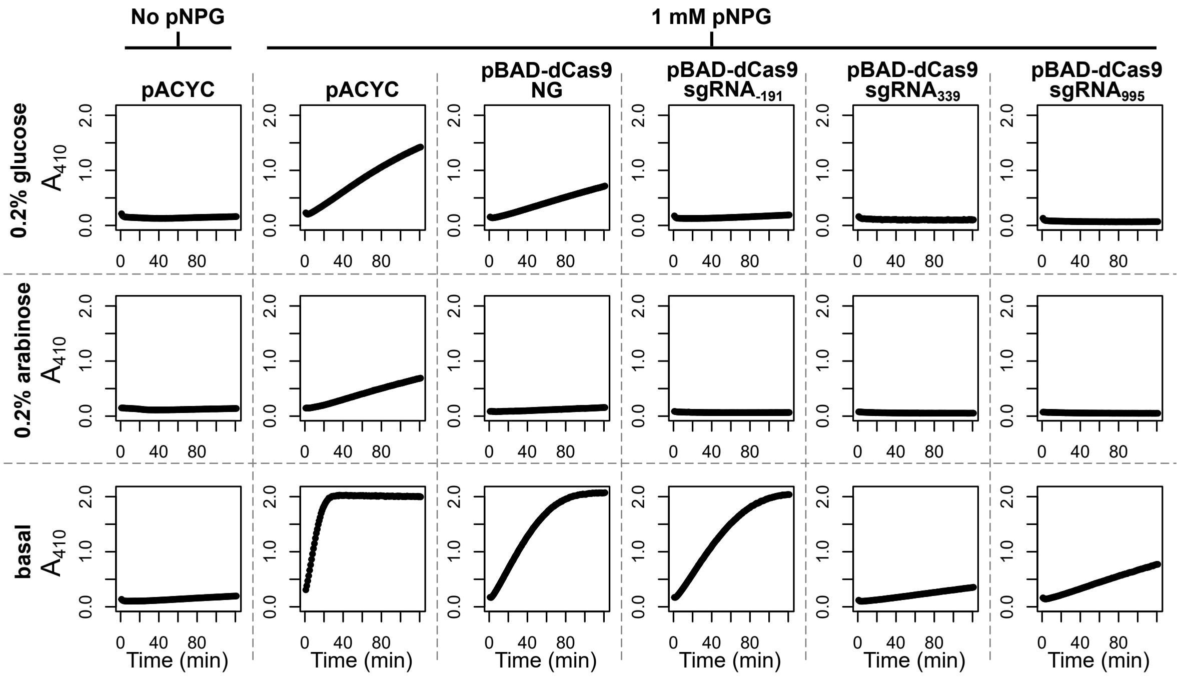

Figure S6: Plots of the absorbance at 410 nm over time in minutes for the GusA chromogenic activity assays, carried out on *E. coli* BL21(DE3) cell extracts that were grown with the indicated plasmids and growth conditions. The leftmost column was grown without pNPG: with 0.2% D-glucose (top row) or 0.2% L-arabinose (middle row) in the media, no GusA activity was detectable in the assay; without glucose or arabinose in the media (bottom row), a small amount of pNPG hydrolysis was detectable. The remaining columns contained 1 mM pNPG in their growth media. Cell growth with glucose or arabinose reduced the amount of detectable pNPG hydrolysis compared to the basal growth conditions, regardless of the presence of the pBAD-dCas9 plasmid. The initially low levels of GusA activity when grown with glucose or arabinose confounded the levels of sgRNA repression. Basal growth conditions were still sufficient to observe GusA repression by sgRNAs and was more effective at distinguishing the variable repression of different sgRNAs.

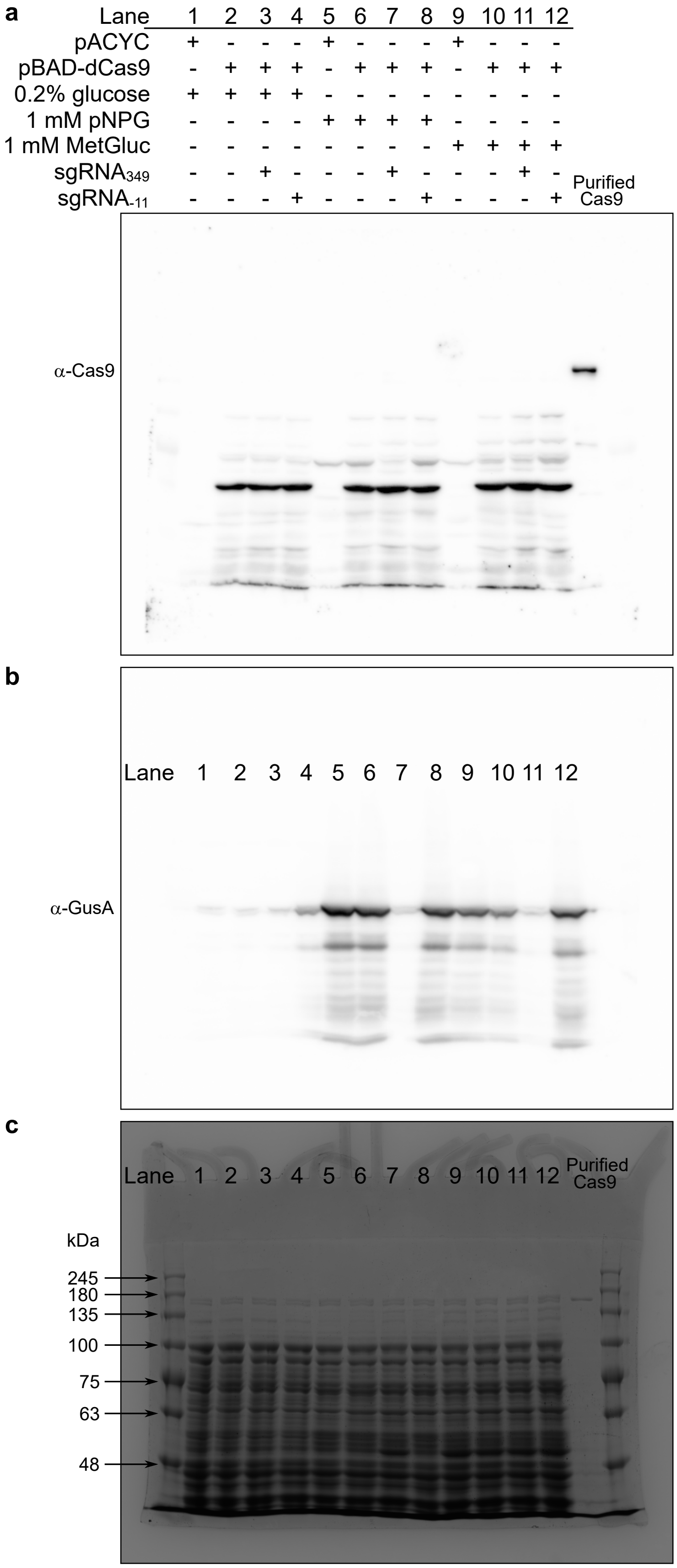

Figure S7: Expression of **a** dCas9 and **b** GusA under the indicated conditions with *E. coli* harbouring the pACYC backbone plasmid or pBAD-dCas9 with no guide, with a good guide ($sgRNA_{349}$), or with a bad guide ($sgRNA_{-11}$) assessed by western blots with a polyclonal anti-Cas9 antibody and a polyclonal anti-GusA antibody, respectively. **c** Raw image of identical protein samples stained with Coomassie blue.

a

	Sequence	Promoter	RBS	CUG start codon
pGreg-GusR-dCas9.0	TTGACGGCTAGCTCAGTCCTAGGTATTGTGCTAGCAATTTAACTTTAATAAGGAGGATTATAATGATG	J23118	Strong	✗
pGreg-GusR-dCas9.1	ATGATAGGCGCTCACCTGACAACGCGGTAAACTAGTTATTCACGCTAACTATAATGGTTTAATGATG	Native	Native	✗
pGreg-GusR-dCas9.2	ATGATAGGCGCTCACCTGACAACGCGGTAAACTAGTTATTCACGCTAACTATAATGGTTTACTGCTG	Native	Native	✓
pGreg-GusR-dCas9.3	TTGACGGCTAGCTCAGTCCTAGGTATTGTGCTAGCAATTTAACTTTAATAAGGAGGATTATACTGCTG	J23118	Strong	✓
pGreg-GusR-dCas9.4	TTGACGGCTAGCTCAGTCCTAGGTATTGTGCTAGCAATTTAACTTTAATACCTATTTCACACATGATG	J23118	Weakest	✗
pGreg-GusR-dCas9.5	TTGACGGCTAGCTCAGTCCTAGGTATTGTGCTAGCAATTTAACTTTAATATCTCCAAATCTGATGATG	J23118	Weak	✗
pGreg-GusR-dCas9.6	TTGACGGCTAGCTCAGTCCTAGGTATTGTGCTAGCAATTTAACTTTAATACCTATTTCACACCTGCTG	J23118	Weakest	✓
pGreg-GusR-dCas9.7	TTGACGGCTAGCTCAGTCCTAGGTATTGTGCTAGCAATTTAACTTTAATATCTCCAAATCTGCTGCTG	J23118	Weak	✓
pGreg-GusR-dCas9.8	CTGATGGCTAGCTCAGTCCTAGGGATTATGCTAGCAATTTAACTTTAATAAGGAGGATTATAATGATG	J23113	Strong	✗
pGreg-GusR-dCas9.9	CTGATAGCTAGCTCAGTCCTAGGGATTATGCTAGCAATTTAACTTTAATAAGGAGGATTATAATGATG	J23112	Strong	✗
pGreg-GusR-dCas9.10	CTGATGGCTAGCTCAGTCCTAGGGATTATGCTAGCAATTTAACTTTAATAAGGAGGATTATACTGCTG	J23113	Strong	✓
pGreg-GusR-dCas9.11	CTGATAGCTAGCTCAGTCCTAGGGATTATGCTAGCAATTTAACTTTAATAAGGAGGATTATACTGCTG	J23112	Strong	✓

Promoter RBS Start codon — -35 — -10

b

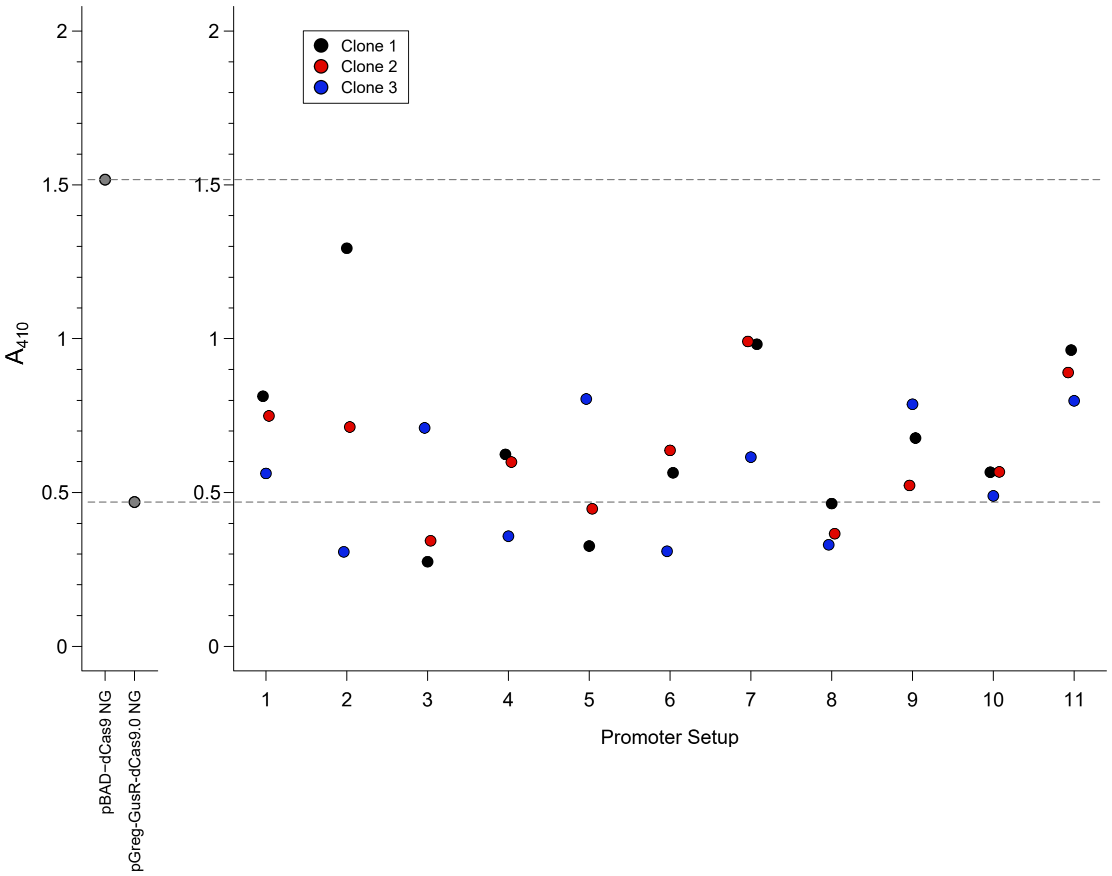

Figure S8: **a** The original GusR promoter (pGreg-GusR-dCas9.0) and 11 alternate promoter designs, with indicated elements highlighted. Fragments were ordered as gBlocks[TM] from IDT with 20-30 bp of homology on both ends to a pGreg-GusR-dCas9.0 PCR product, and were then cloned with the NEBuilder® HiFi DNA assembly kit. Assemblies for each of the 11 alternate promoter pGreg-GusR-dCas9 plasmids were transformed into $CaCl_2$-competent *E. coli* cells. **b** Left panel, *E. coli* with the pBAD-dCas9 NG plasmid and the pGreg-GusR-dCas9.0 NG plasmid as positive and negative controls for GusA activity, respectively, were 1:50 diluted from saturated cultures into selective LB supplemented with 1 mM pNPG. A_{410} was monitored as cultures grew in the BioTek Epoch 2 microplate spectrophotometer to use pNPG cleavage as a measure of GusA activity. The endpoint measurement at 3.5 hours is shown. Right panel, 3 colonies from each promoter setup assembly transformation were grown and monitored in the BioTek Epoch 2 microplate spectrophotometer alongside the controls. The endpoint A_{410} measurement at 3.5 hours is shown for each clone. Clones with $A_{410} \geq 0.25$ units higher than pGreg-GusR-dCas9.0 were sent for Sanger sequencing verification. Some clones had mutations in the promoter elements, potentially recovering GusA activity by no longer expressing the plasmid-encoded GusR. Sequence-verified constructs from promoter setups 1, 7, and 11 were examined further.

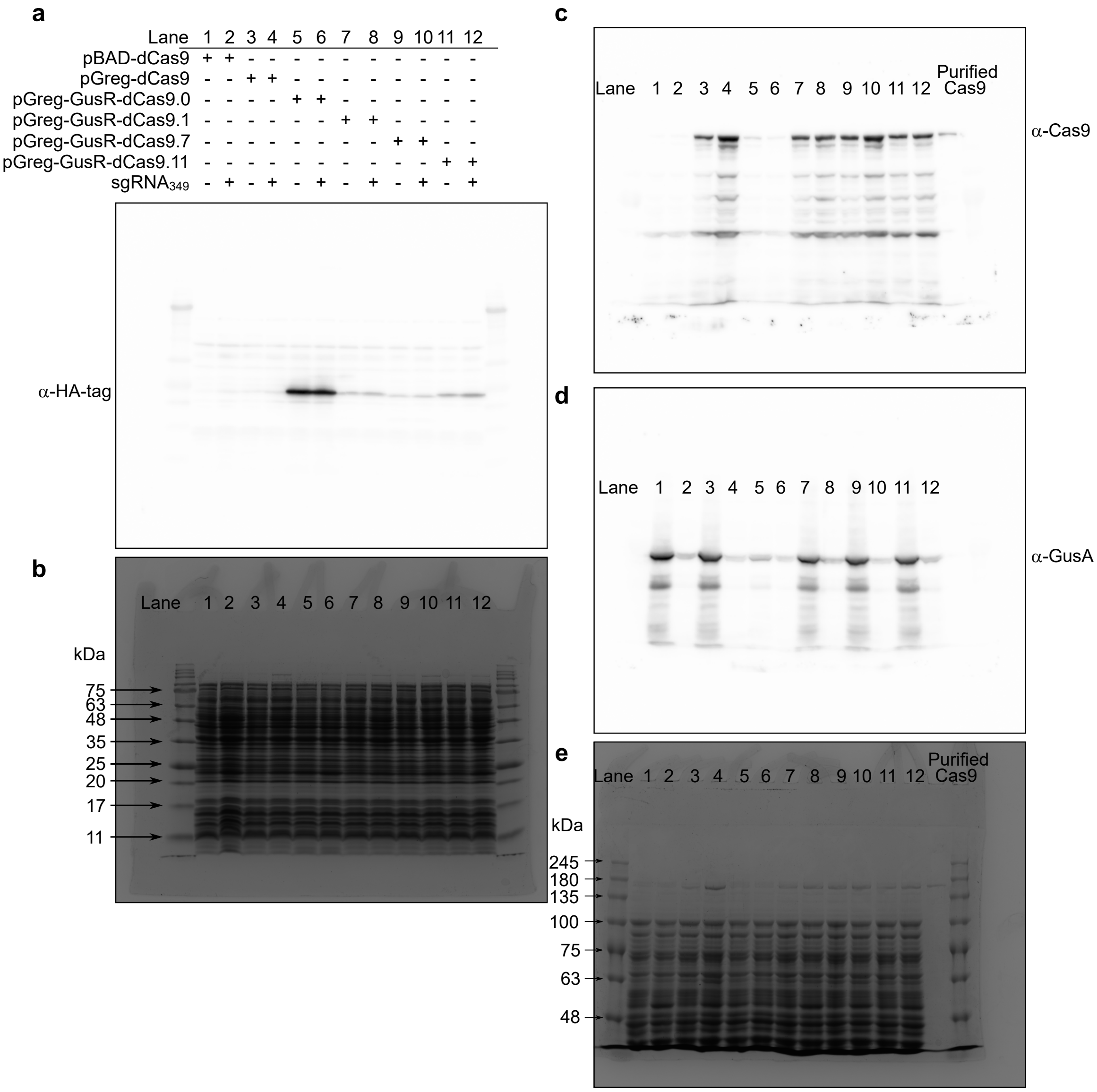

Figure S9: Raw images associated with figure 4C. **a** Expression of GusR with the indicated promoter systems with *E. coli* harbouring the pGreg-GusR-dCas9 plasmid grown in selective media supplemented with 1 mM pNPG assessed by western blots with a monoclonal anti-HA-tag antibody. **b** Raw image of identical protein samples on a 15% SDS-PAGE gel stained with Coomassie blue. **c** Expression of dCas9 from identical protein samples assessed by western blots with a polyclonal anti-Cas9 antibody. **d** Expression of GusA from identical protein samples assessed by western blots with a polyclonal anti-GusA antibody. **e** Raw image of identical protein samples on an 8% SDS-PAGE gel stained with Coomassie blue.

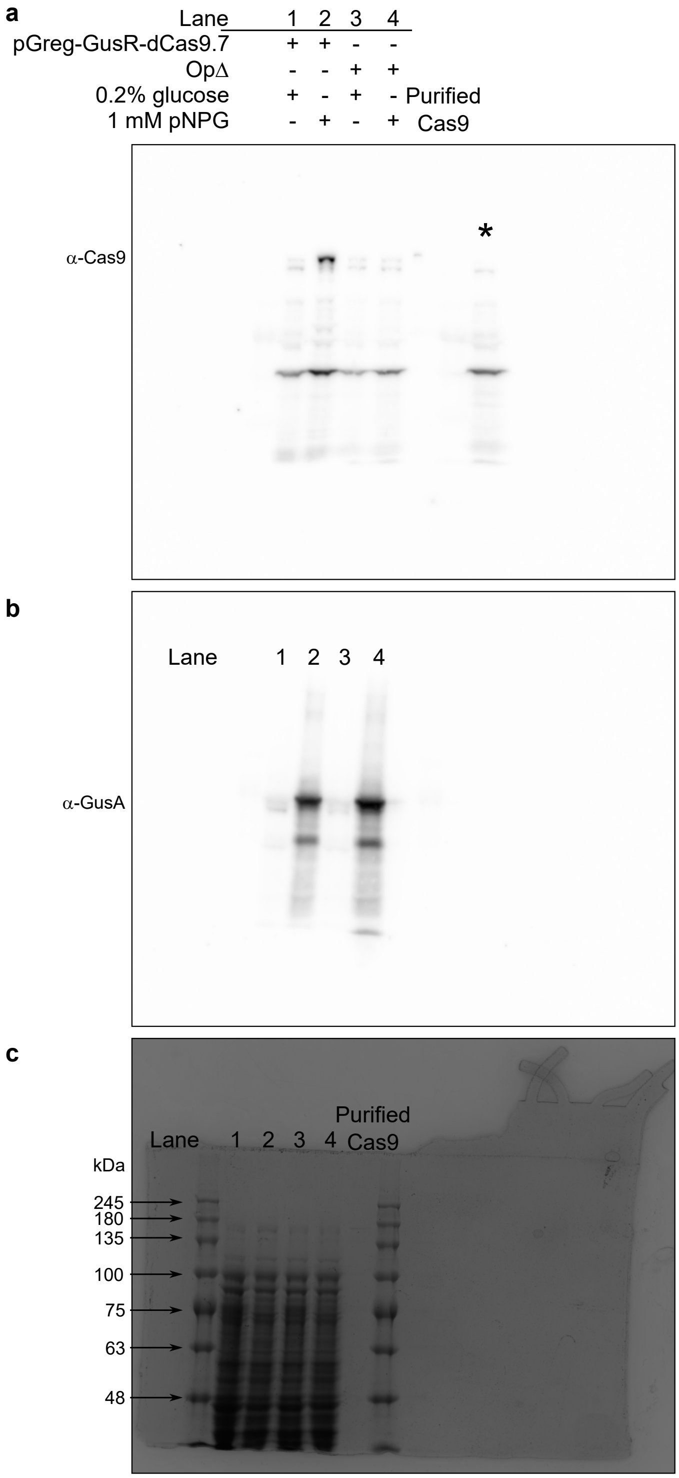

Figure S10: Raw images associated with figure 4D. Expression of **a** dCas9 and **b** GusA grown under the indicated conditions with *E. coli* harbouring the pGreg-GusR-dCas9.7 plasmid or the OpΔ plasmid with no guide assessed by western blots with a polyclonal anti-Cas9 antibody and a polyclonal anti-GusA antibody, respectively. *Lane 1 was loaded a second time on the gel that was transferred and blotted with the anti-Cas9 antibody. **c** Raw image of identical protein samples stained with Coomassie blue.

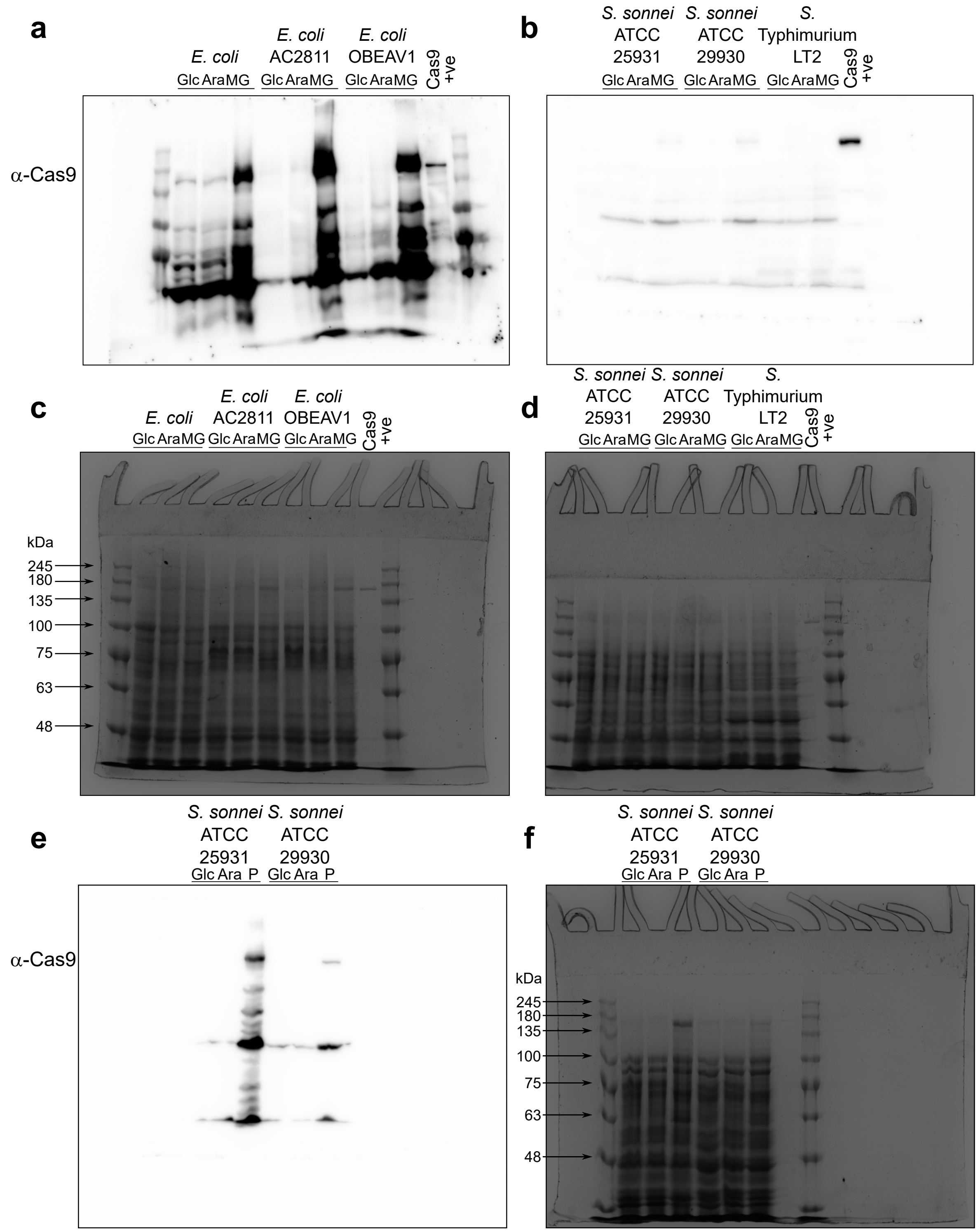

Figure S11: Raw images associated with figure 5B. **a** and **b** Expression of dCas9 in the indicated strains with pGreg-GusR-dCas9.7 grown in LB supplemented with 0.2% glucose (Glc), 0.2% arabinose (Ara), or 1 mM methyl β-D-glucuronide sodium salt (MG) assessed by western blots with a polyclonal anti-Cas9 antibody. **c** and **d** Raw image of identical protein samples stained with Coomassie blue. **e** Expression of dCas9 in the indicated *S. sonnei* strains with pGreg-GusR-dCas9.7 grown in LB supplemented with 0.2% glucose (Glc), 0.2% arabinose (Ara), or 1 mM pNPG (P) assessed by western blots with a polyclonal anti-Cas9 antibody. **f** Raw image of identical protein samples stained with Coomassie blue.

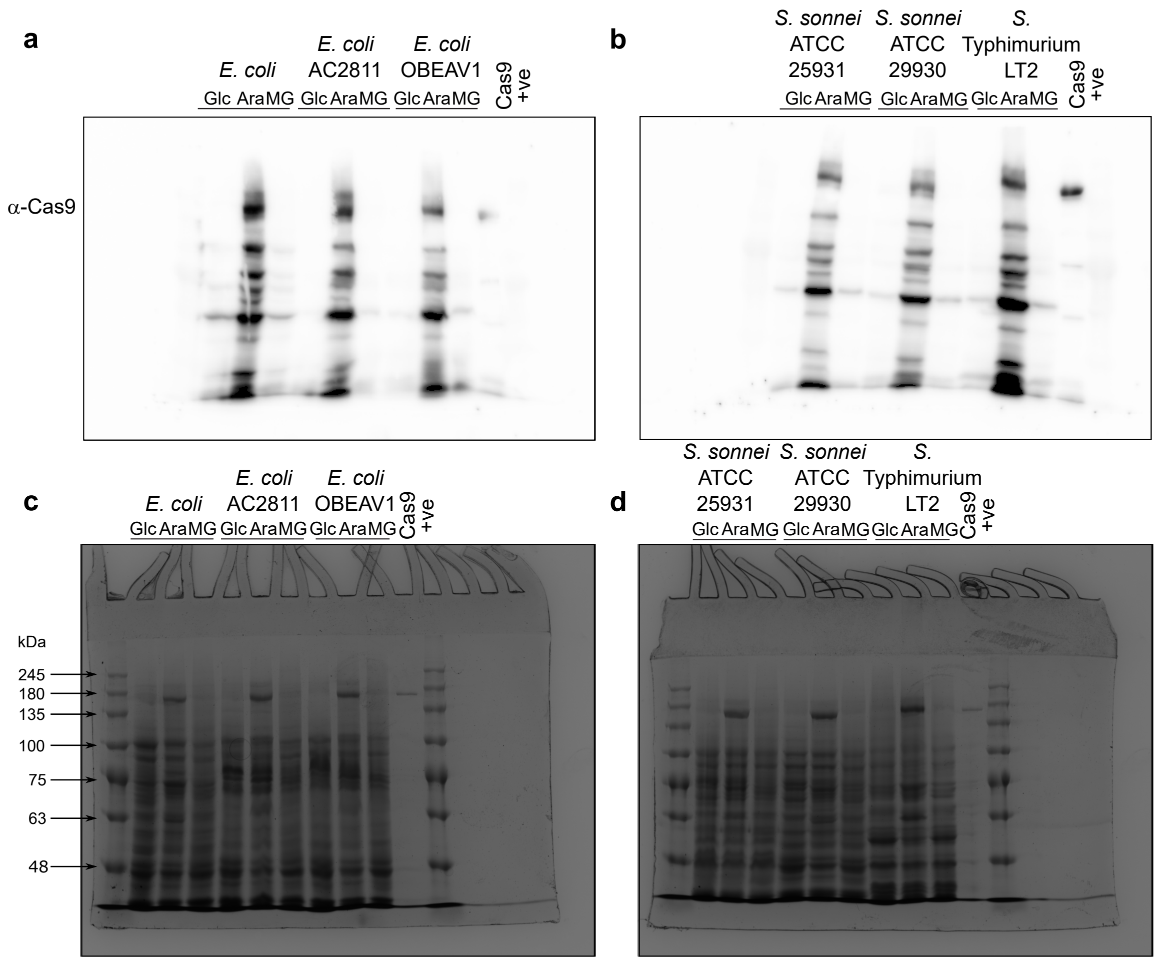

Figure S12: Raw images associated with figure 5C. **a** and **b** Expression of dCas9 in the indicated strains with pBAD-dCas9 grown in LB supplemented with 0.2% glucose (Glc), 0.2% arabinose (Ara), or 1 mM methyl β-D-glucuronide sodium salt (MG) assessed by western blots with a polyclonal anti-Cas9 antibody. **c** and **d** Raw image of identical protein samples stained with Coomassie blue.

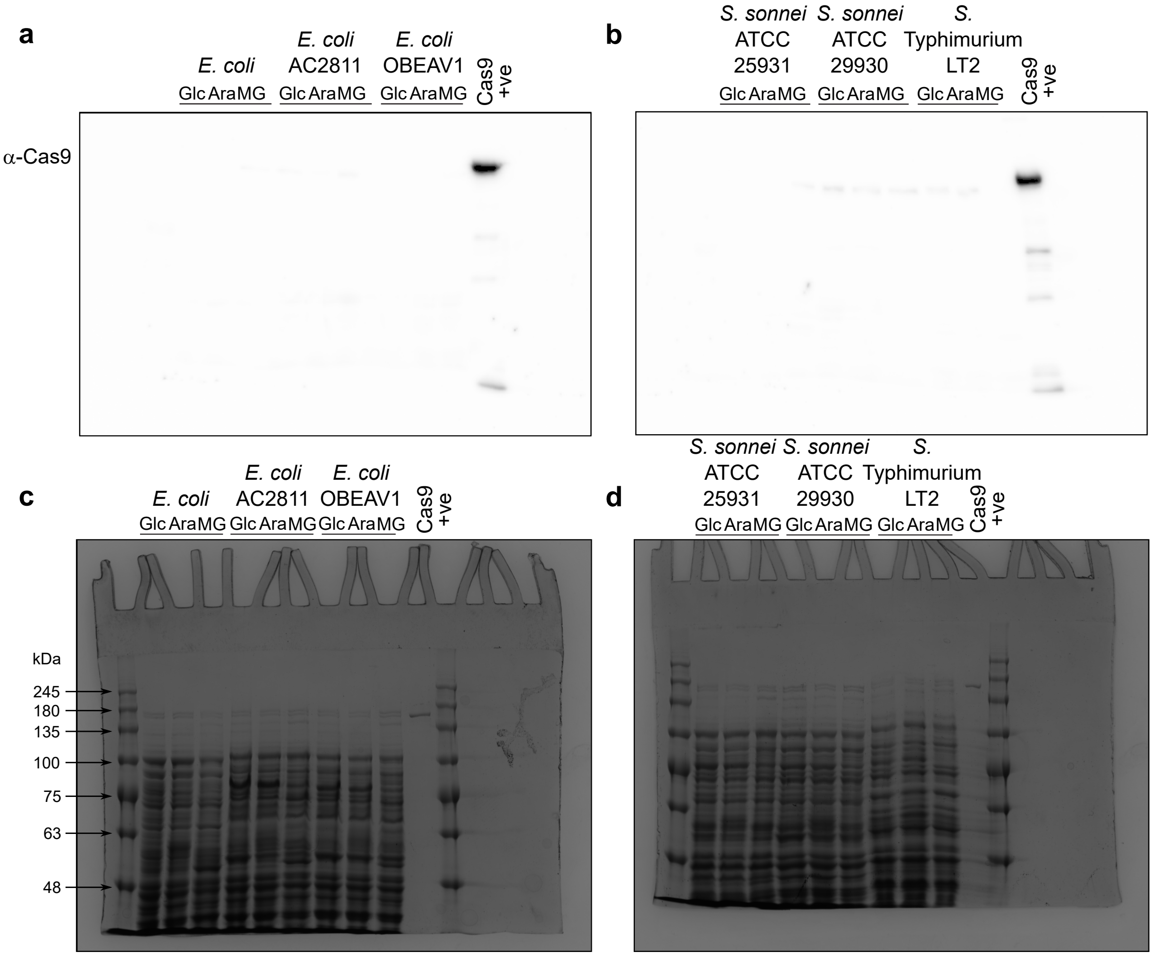

Figure S13: Raw images of microbiome species without a dCas9 plasmid blotted for Cas9. **a** and **b** Expression of dCas9 in the indicated strains with no dCas9 plasmids grown in LB supplemented with 0.2% glucose (Glc), 0.2% arabinose (Ara), or 1 mM methyl β-D-glucuronide sodium salt (MG) assessed by western blots with a polyclonal anti-Cas9 antibody. **c** and **d** Raw image of identical protein samples stained with Coomassie blue.

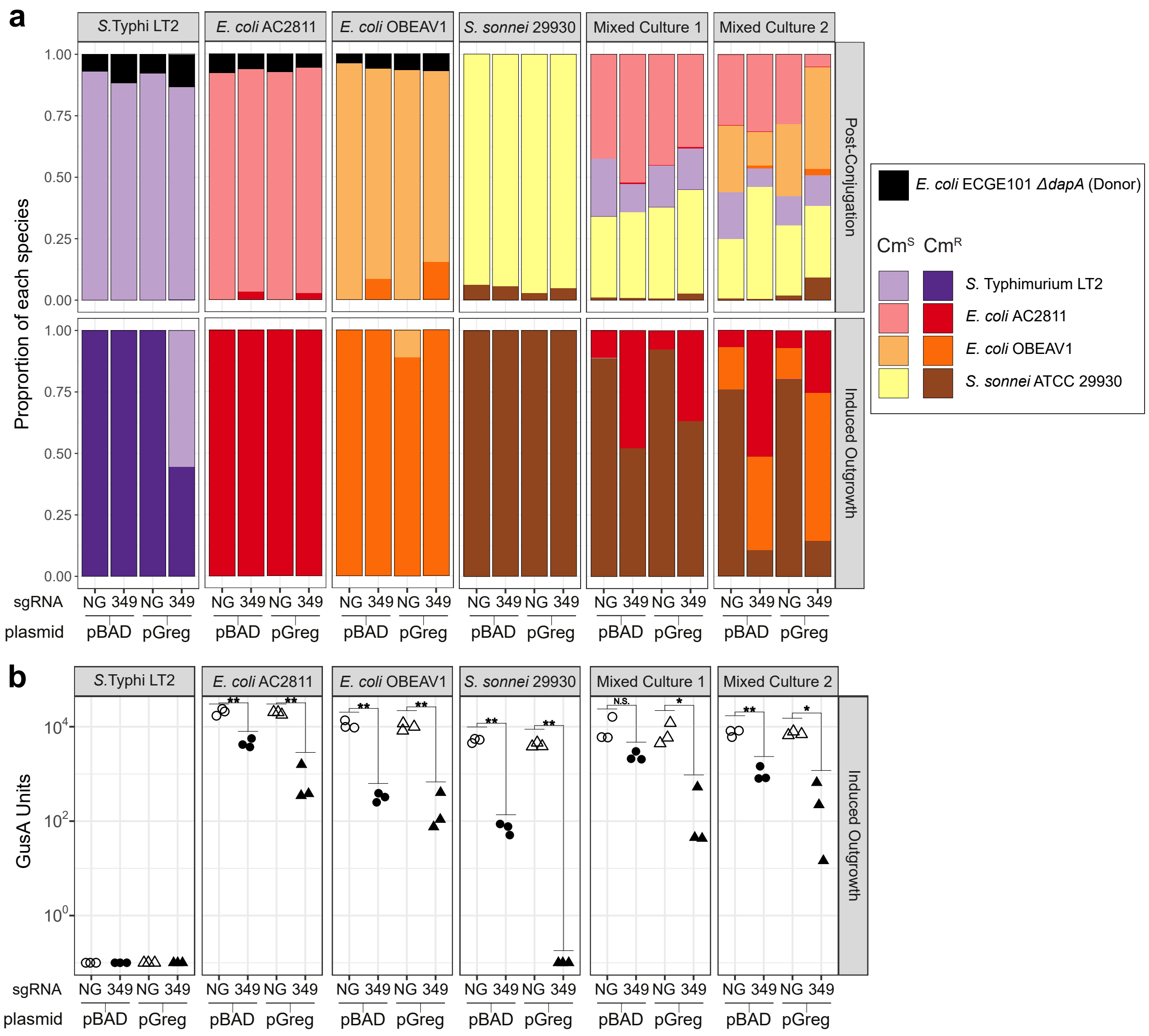

Figure S14: Repression of GusA activity in a mixed community. **a** Mean proportions from 3 biological replicates of each species in the single or mixed recipient cultures calculated from CFU/mL following conjugation of pBAD-dCas9 (pBAD) or pGreg-GusR-dCas9.7 (pGreg) without (NG) or with $sgRNA_{349}$. **b** GusA activity units from single and mixed recipient cultures following conjugation of pBAD-dCas9 (circles) or pGreg-GusR-dCas9.7 (triandgles) without (open data points) or with $sgRNA_{349}$ (filled data points). GusA units from three biological replicates are shown. N.S. not significant, *$P < 0.05$, **$P < 0.005$ calculated by t-test.
