# Supplementary material for: Metabolically-targeted dCas9 expression in bacteria
Source: Nucleic Acids Res. 2023 Jan 11;51(2):982–96. doi: 10.1093/nar/gkac1248 (PMC9881133; doi:10.1093/nar/gkac1248)
Supplement: gkac1248_Supplemental_Files [file gkac1248_supplemental_files.zip › Supplementary_table_legends.pdf]

Supplementary Table Legends.

Supplementary Table S1. sgRNAs used in this study. GusA units and St.Dev are the mean and standard deviation, respectively, of three technical replicates of the chromogenic GusA activity assay. Target position indicates where the sgRNA is targeting dCas9 to bind, in nucleotides relative to the first coding nucleotide of the *gusA* gene. Target strand indicates if the sgRNA is targeting the nontemplate (NT) or template (T) strand. Top Strand (5`-3`) sequence of sgRNA is the targeting crRNA sequence of the sgRNA, complementary to a target sequence upstream of or within the *gusA* gene.

Supplementary Table S2. Results of RNA-Seq analysis. Sheet 1 (Trimmed and Mapped Counts) shows the counts used for differential expression analysis from each of the three biological replicates for no guide (NG), $sgRNA_{349}$, $sgRNA_{373}$, $sgRNA_{451}$, and $sgRNA_{980}$. Sheets 2-5 show the output from DeSeq2 analysis between NG and each sgRNA. The gene ID indicates the gene in the BL21(DE3) gff3 annotated genome (https://github.com/tbrowne5/Metabolically-targeted-dCas9-expression-in-bacteria-.git); the baseMean is the mean of the normalized count values over all samples; the log2FoldChange is the effect size estimate, indicating how much a transcripts expression has changed between the no guide and with guide condition; the lfcSE is the standard error of the log2 fold change; stat is the Wald statistic from the Wald test; pvalue is the p-value from the Wald test; and padj is the Benjamini-Hochberg adjusted p-value from the Wald test.

Supplementary Table S3. Primers and oligonucleotides used in this study. The notes column describes how each primer or oligonucleotide was used.
